# SegVeg: Segmenting RGB Images into Green and Senescent Vegetation by Combining Deep and Shallow Methods

**DOI:** 10.34133/2022/9803570

**Published:** 2022-10-11

**Authors:** Mario Serouart, Simon Madec, Etienne David, Kaaviya Velumani, Raul Lopez Lozano, Marie Weiss, Frédéric Baret

**Affiliations:** ^1^Arvalis, Institut du végétal, 228, route de l'aérodrome - CS 40509, 84914 Avignon Cedex 9, France; ^2^INRAE, Avignon Université, UMR EMMAH, UMT CAPTE, 228, route de l'aérodrome - CS 40509, 84914 Avignon Cedex 9, France; ^3^CIRAD, UMR TETIS, F-34398 Montpellier, France; ^4^Hiphen SAS, 228, route de l'aérodrome - CS 40509, 84914 Avignon Cedex 9, France

## Abstract

Pixel segmentation of high-resolution RGB images into chlorophyll-active or nonactive vegetation classes is a first step often required before estimating key traits of interest. We have developed the SegVeg approach for semantic segmentation of RGB images into three classes (background, green, and senescent vegetation). This is achieved in two steps: A U-net model is first trained on a very large dataset to separate whole vegetation from background. The green and senescent vegetation pixels are then separated using SVM, a shallow machine learning technique, trained over a selection of pixels extracted from images. The performances of the SegVeg approach is then compared to a 3-class U-net model trained using weak supervision over RGB images segmented with SegVeg as groundtruth masks. Results show that the SegVeg approach allows to segment accurately the three classes. However, some confusion is observed mainly between the background and senescent vegetation, particularly over the dark and bright regions of the images. The U-net model achieves similar performances, with slight degradation over the green vegetation: the SVM pixel-based approach provides more precise delineation of the green and senescent patches as compared to the convolutional nature of U-net. The use of the components of several color spaces allows to better classify the vegetation pixels into green and senescent. Finally, the models are used to predict the fraction of three classes over whole images or regularly spaced grid-pixels. Results show that green fraction is very well estimated (*R*^2^ = 0.94) by the SegVeg model, while the senescent and background fractions show slightly degraded performances (*R*^2^ = 0.70 and 0.73, respectively) with a mean 95% confidence error interval of 2.7% and 2.1% for the senescent vegetation and background, versus 1% for green vegetation. We have made SegVeg publicly available as a ready-to-use script and model, along with the entire annotated grid-pixels dataset. We thus hope to render segmentation accessible to a broad audience by requiring neither manual annotation nor knowledge or, at least, offering a pretrained model for more specific use.

## 1. Introduction

The vegetation fraction (VF) is a key trait that drives the partitioning of radiation between the background and the vegetation. It is used in several studies as a proxy of crop state [1] and yield [2, 3]. The complement to unity of VF is the gap fraction that is used to estimate the plant area index. However, several ecophysiological processes such as photosynthesis and transpiration are driven by the amount of green surfaces that exchange mass and energy with the atmosphere. More specifically, the green fraction (GF) is used to estimate the green area index (GAI) [4] defined as the area of green vegetation elements per unit horizontal ground area. GF is a more relevant trait that should be used when describing crop functioning [5]. The difference between VF and GF is the senescent fraction (SF = VF − GF), sometimes called the nonphotosynthetic fraction [6, 7]. For crops, SF depends on both the growth stage and state of the plants. The SF trait is used to characterize a biotic or abiotic stress, describe nutrient recycling, and monitor the ageing process [8–10]. Some studies have demonstrated the ability of genotypes to stay green by delaying senescence and potentially improve productivity [11, 12].

Several remote sensing methods have been developed to estimate GF and SF using the spectral variation of the signal observed at the canopy scale from metric to decametric resolution [13]. VF, GF, and SF can be also computed using very high spatial resolution images with pixel sizes from a fraction of mm to cm, i.e., significantly smaller than the typical dimension of the objects (plants, organs). RGB cameras with few to tens of millions of pixels are currently widely used as noninvasive high-throughput techniques applied to plant breeding, farm management, and yield prediction [14–16]. These cameras are borne on multiple platforms, including drones [17], ground vehicles [18], and handheld systems [19], or set on a fixed pod [16].

Several methods have been proposed to identify the green pixels in RGB images including thresholding color indices [20] and machine learning classification [21] based on few color space representations. However, these techniques are limited at least by one of the two main factors:

- Confounding effects: depending on the illumination conditions and on the quality of the camera optics, part of the soil may appear green due to chromatic aberration. Further, parts of the image that are saturated, with strong specular reflection or very dark, will be difficult to classify using only the color of the pixel. Finally, the soil may also appear greenish when it contains algae [22]
- Continuity of colors: at the cellular scale, senescence results from the degradation of pigments that generally precedes cell death [23]. During the degradation process, changes in the pigment composition result into a wide palette of leaf color in RGB imagery, with a continuity between “green” and “senescent” states. Further, when pixels are located at the border of an organ, its color will be intermediate between organ and background. This problem is obviously enhanced when the spatial resolution of the RGB image is too coarse

It is therefore difficult to segment accurately and robustly the green vegetation parts of a RGB image using only the color information of pixels. Same limitations apply to the segmentation of the senescent vegetation parts. In addition, crop residues located in background areas are difficult to distinguish from the senescent vegetation observed on standing plants with very similar range of brownish colors. Textural and contextual information should therefore be exploited to better segment RGB images into green and senescent vegetation parts.

Semantic segmentation [24] that assigns a class to each pixel of the image appears to be an attractive approach. It is based on deep learning techniques and has been applied to several domains including urban scene description for autonomous vehicles, medical imagery [25], and agriculture [26, 27]. However, images need to be labelled exhaustively into several target classes, which requires large annotation resources [28].

The objective is then to develop and evaluate a two-step semantic segmentation approach called SegVeg. It labels each pixel of very high-resolution RGB images of vegetation scenes into three classes: background, green, and senescent vegetation. It has been designed to reduce the annotation effort by combing a convolutional neural network (CNN) that splits image into vegetation (including both green and senescent pixels) and background, to a simple support vector machine (SVM) technique that classifies the vegetation pixels into green and senescent. SegVeg will be compared to a CNN classifier that directly identifies background, green, and senescent vegetation pixels following a weak supervised training principle.

## 2. Materials and Methods

**Figure 1:**
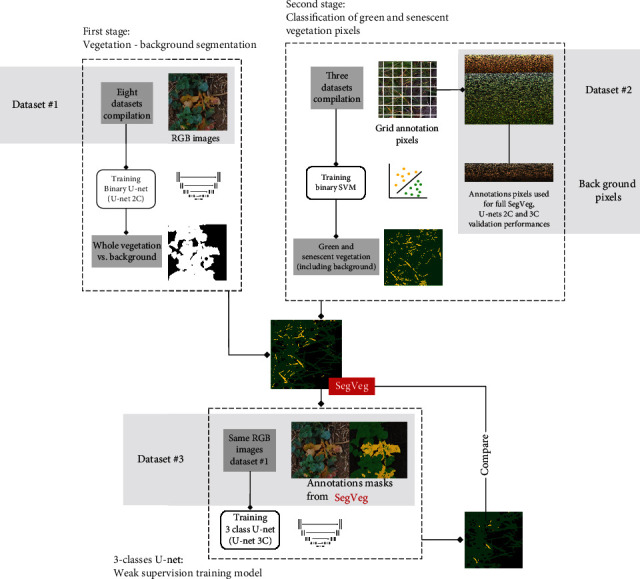
Flowchart describing the overall approach of the study.

As shown in Figure 1, this study investigates two approaches to segment images in three classes: green, senescent, and background.

The first step consists of developing the SegVeg method that combines a binary U-net model (U-net 2C) to first separate vegetation from background. Then a SVM model will separate green from senescent vegetation, once the whole vegetation is extracted.

This stage relies on two training datasets: fully annotated patches (Dataset #1 with 2-class entire masks) for the U-net 2C training and pixel labelled datasets for the SVM approach (Dataset #2). Once the SegVeg approach is set, it is used to build a third dataset of fully nonsupervised annotated patches (Dataset #3) and train a 3-class U-net model (U-net 3C) on the same RGB images present in Dataset #1. The SegVeg and 3-class U-net performances are then compared.

### 2.1. The SegVeg Approach

**Figure 2:**
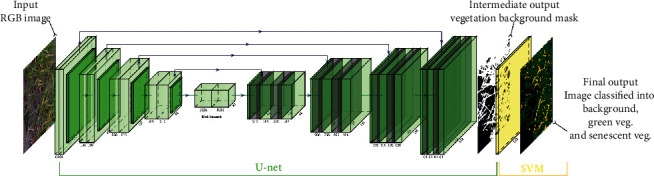
Illustration of the SegVeg architecture inputs and outputs. The first stage is a U-net model that predicts vegetation and background masks. The second stage is a SVM that classifies the vegetation mask into green and senescent pixels. The two stages were trained over two independent datasets.

The SegVeg approach is made of two stages (Figures 1 and 2). In the first stage, the whole image is classified into vegetation and background mask using a U-net type deep learning network [29]. Then, vegetation pixels (predicted from the first stage) are classified into green and senescent vegetation using a SVM. The two binary outputs of each model are then merged to form a 3-class mask.

#### 2.1.1. First Stage: Vegetation and Background Segmentation

U-net is a deep learning model with encoder-decoder network architecture that is widely used for image semantic segmentation. The model was trained over the labelled images from Dataset #1 (Section 2.3.1) to predict two classes: vegetation (green and/or senescent) and background. EfficientNet-B2 architecture [30] with weights initialized on ImageNet was used as the backbone architecture. Patches of 512 × 512 pixels were used for training after data augmentation based on the Albumentations library [31]. The training process was based on a Dice loss function with an Adam optimizer.

A predefined decaying learning rate schedule (step based) was used to reach local minima, with an initial value of 0.01 and reaching at the end 10*e* − 6, which is an usual range in standard multilayer neural networks studies [32]. The minibatch size was set to 32 for computational purpose. Finally, early stopping was implemented to set number of training iterations. The Python Segmentation Models library under PyTorch was used [33] with GPU activation (GeForce RTX 3090).

#### 2.1.2. Second Stage: Classification of Green and Senescent Vegetation Pixels

The support vector machine (SVM) is an efficient machine learning classification method widely used for image segmentation [34–36]. It maps the original features to some higher-dimensional space where the training dataset is separable. Several color spaces and transformations [37] were used to classify green and senescent pixels including RGB, HSV, CIELab, grayscale, luminances, CMYK, YCbCr, and YIQ derived from the original RGB values. A total of 23 potential input features were thus computed, namely R, G, and B; H, S, and V; L, a, and b; GE; LA, LB, and LC; C, M, Y, and K; Yi, Cb, and Cr; and Yj, I, and Q. However, the possible redundancy and irrelevancy of some features may decrease the accuracy of the classification. We then selected the most appropriate inputs using the step forward wrapper method [38]. Finally, 14 input features were retained: R, G, B, H, S, a, b, GE, M, YE, Cb, Cr, I, and Q.

This second-stage SVM was 4calibrated over labelled pixels from Dataset #2 (see Section 2.3.2). The hyperparameters were tuned using a grid search algorithm following a leave-one-out cross-validation principle. This process led to the optimal values *C*: 1 and *γ*: 10^−3^, and kernel *rbf* was set according to prior knowledge that data are not linearly separable. Scikit 0.23.2 with Python 3.7 was used for implementation [39].

### 2.2. The 3-Class U-net Model (U-net 3C)

A three-class U-net model was used as a reference to evaluate the proposed SegVeg approach (Figure 1).

However, due to the unavailability of a dataset containing entire images annotated into three classes (background, green, and senescent vegetation), we prepared 3-class masks by applying SegVeg over the RGB images used to train U-net 2C (i.e., Dataset #1). Indeed, to reduce the annotation effort, the second-stage SVM was trained over pixels extracted from regularly spaced grids, explained in the following dataset sections. Therefore, no manually annotated 3-class masks were available as groundtruth references.

The same U-net architecture and hyperparameters used for U-net 2C of the SegVeg approach were also employed here during training.

### 2.3. Training and Testing Datasets

#### 2.3.1. Dataset #1: Vegetation and Background Fully Annotated Patches

Eight subdatasets from previous studies were compiled to get a wide range of acquisition conditions, species, crop states, and stages (Table 1).

**Figure 3:**
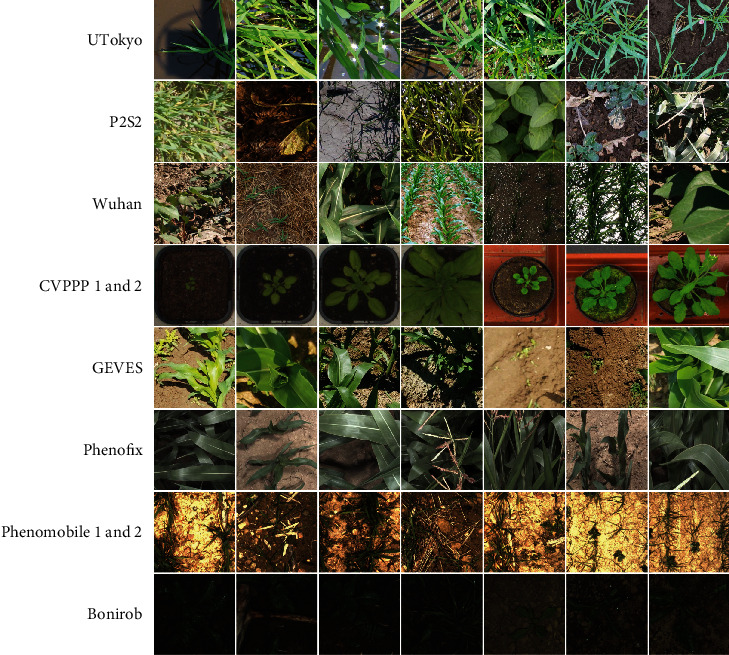
Sample of 512 × 512 pixels patches extracted from the eight subdatasets (Dataset #1).

The images were acquired with several cameras equipped with different focal length optics and variable distances from the ground. All blurred images or those with poor quality were excluded from our study. The original images were then split into several square patches of 512 × 512 pixels, a size selected to keep sufficient context. A total of 2015 patches were extracted, showing a large diversity as illustrated in Table 2. The ground sampling distance (GSD) ranges were between 0.3 and 2 mm to capture enough details (Figure 3).

Considering that image annotation is time consuming, it was subcontracted to a private company, imageannotation.ai. Each original image was carefully segmented by several operators into vegetation (green and senescent combined) and background pixels. We then verified the resulting classified images and reannotated the few wrongly annotated ones.

#### 2.3.2. Dataset #2: Green, Senescent, and Background Annotated Pixels

Dataset #2 is composed of annotated pixels only, extracted from images on which we have affixed regular square matrix (grids) of 8 to 11 pixels.

This dataset was used to train and test the SVM stage of the SegVeg approach (on green and senescent pixels). After adding the background pixels, it was also used to evaluate the performances of both the SegVeg and the U-nets (2C and 3C).

*(1) Image Acquisition and Extraction*. Three independent datasets (LITERAL, PHENOMOBILE, and P2S2) were used to train and evaluate the proposed methods.

**Figure 4:**
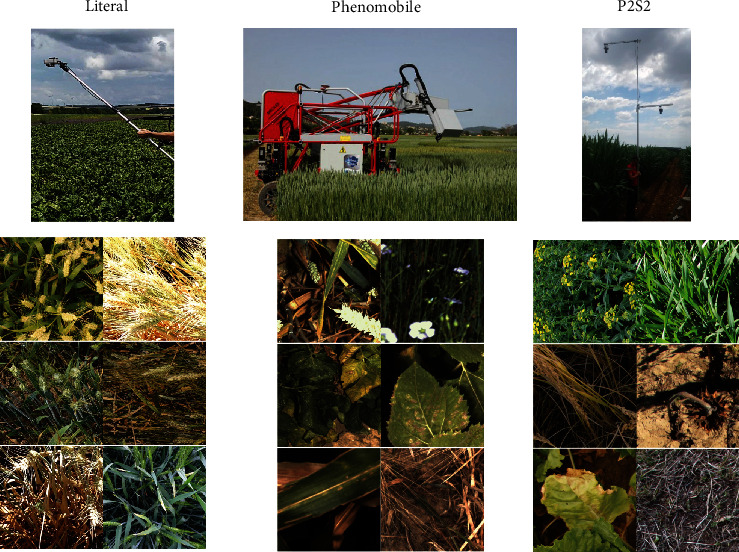
The acquisition systems used for the three independent datasets in Dataset #2: LITERAL, PHENOMOBILE, and P2S2 and their respective examples of 512 × 512 images patches extracted from the three systems.

- The LITERAL dataset was acquired with a handheld system called LITERAL (Figure 4). An operator maintains a boom with a Sony RX0 camera fixed at its extremity. The camera faced the ground from nadir at an approximately fixed distance (Table 3). The 68 available annotated images covered a wide range of wheat genotypes grown at several locations in France, representing different growth stages, soil backgrounds, and illumination conditions
- The PHENOMOBILE dataset was acquired with the Phenomobile system (Figure 4), an unmanned ground vehicle [46]. This system uses flashes for image acquisition making the measurements independent of the natural illumination conditions. Images are acquired from nadir at a fixed distance from the top of the canopy (Table 3). The 173 available annotated images covered six crops grown in four phenotyping platforms in France (Table 3)
- The P2S2 dataset is composed of 200 hemispherical and nadir images. The acquisition was designed to provide a large dataset over a wide range of crops, observed under contrasted growth conditions, throughout the crop growth cycle, covering crucial phenological stages. More details on the dataset can be found in [42]

Several cameras were used for the acquisition of the three datasets, resulting in differences in image quality and GSD (Table 4). Note that the GSD of this dataset (Table 4) is consistent with that of the previous dataset (Table 2). A total of 441 images of 512 × 512 pixels were finally selected to represent a wide diversity (Figure 3).

*(2) Pixel Labelling*. The previously mentioned pixel grids were classified into one of the following six classes, namely, *green vegetation*, *senescent vegetation*, *background*, *green/senescent vegetation unsure*, *unknown*, and *others*. This allowed us to remove pixels with uncertain annotations and potential bias in the training phase. The *green/senescent vegetation unsure*, *unknown*, and *others* were for instance not used in the training and evaluation of the proposed models. However, because of the complexity, subjectivity, and time required to assign pixels into the six classes listed above, the annotation was limited to a small number of pixels per patches (i.e., not building full 3-classes groundtruth masks). This sampled annotation is possible because the second stage of SegVeg (shallow machine learning SVM method) does not require context or local information and therefore not demanding entire patches to be exhaustively annotated. We used a grid displayed on each 512 × 512 images, where the pixels to be classified were located at the intersection of the grid points. A video recording the annotation process of a few pixels is available in Supplementary Material (figure S1). The regular square matrix can vary from 8 to 11 pixels on a side, depending on images. The web based platform, Datatorch [47], was used by 2 annotators. A second round of pixel labelling was performed by 2 other reviewers to find a better consensus on the uncertain pixels and to avoid potential bias in building Dataset #2.

Among the 441 annotated images (Table 4), the unsure classes represented about 16% of the total number of pixels. It can be noticed that for the PHENOMOBILE dataset, the use of integrated flashes during image acquisition provided better pixel interpretation leading to fewer confusions. This dataset is publicly available on Zenodo and can be accessed by following the guidelines at this link https://github.com/mserouar/SegVeg.

*(3) Split between Training and Testing Datasets*. A total of 19,738 pixels were finally available to perform the training and testing of the SegVeg SVM stage, of which 6132 were used for training and 13,606 for testing (Table 5). Note that for the evaluation of U-net approaches (2C and 3C), the test Dataset #2 evolves by adding the almost 6000 background pixels annotated from the grid (Figure 1, Supplementary Material figure S1), which are naturally absent in the green/senescent SVM training and evaluation.

The LITERAL dataset that represented only a small fraction of the available patches over wheat crops was kept entirely for testing. The PHENOMOBILE dataset was split randomly into training (30%) and testing (70%) dataset (Table 5), resulting in 1803 pixels used to train the SVM model. Similarly, P2S2 was randomly split into 4329 pixels for training (about 40%) and the remaining for testing. This allows to get a balanced distribution between the contributions of PHENOMOBILE and P2S2 datasets to the training process as well as maintain a balanced Green/Senescent pixels fraction. The splitting scheme was chosen according to the concrete theoretical foundation of the SVM algorithm. SVMs are usually not chosen for large-scale data studies because their training complexity is highly dependent on the dataset size (quadratic to the number of observations), which also comes with calculation time issues [48–50]. Moreover, the concept of hyperplane and margins does not require a lot of observations during training, and adding observations could lead to poor generalisation properties. A big amount of initial data was hence kept for the validation step, to ensure robustness in predictions and model performances.

### 2.4. Evaluation Metrics

Since semantic segmentation classifies each individual pixel, three standard classification metrics derived from the confusion matrix were used to quantify the performances of the methods at the class level: precision, recall, and *F*1-score (Table 6). Further, the overall accuracy and overall *F*1-score were also computed to get a more global evaluation of the segmentation performances (Table 6). We also considered the fraction of pixels of a certain class in an image in a given viewing direction. This trait is widely used as a proxy of crop development [51] particularly for the green parts characteristic of the photosynthetically active elements [52]. Finally, regression results RMSE and *R*^2^ were also considered to evaluate the methods. All these metrics were computed over the test dataset (Table 5), either directly on the test pixels from the image grids, for grid canopy fractions directly on image grids from which the training pixels have been removed, or finally, on the whole images for U-net 3C step.

## 3. Results

### 3.1. Performances of the SegVeg Approach

#### 3.1.1. Separation of Vegetation | Background with First-Stage U-net 2C Model

Results (Table 7) on background and combined green/senescent vegetation pixel grids show that U-net 2C first-stage model classifies well the vegetation from the background pixels, with an overall mean *F*1-score between 82% and 92%. The *F*1*class* values are higher for the vegetation class. Misclassifications are observed when either the background corresponding to algae/moss is classified as vegetation (Supplementary Material figure S2, bottom) or senescent vegetation is confounded with crop residues (Supplementary Material figure S2, top). The P2S2 subdatasets, achieved the best *F*1_*all*_ performances.

#### 3.1.2. Green and Senescent Vegetation Classification Performances of the SVM Only and Full SegVeg Approach

The pixel classification performances were evaluated on the following: (i) applying only the second-stage SegVeg SVM and (ii) applying the full SegVeg approach. Results (Table 8) show that the green vegetation pixels are generally well identified for the three subdatasets.

When using only the SVM, the senescent vegetation pixels show significant confusion with the green vegetation for the LITERAL subdataset. The background pixels are preferentially classified as senescent vegetation, except for the LITERAL subdataset (in SVM rows Table 8). This highlights the importance of separating first the vegetation from the background with the U-net 2C model since without using contextual information, e.g., using only the RGB color information, does not allow to separate well the vegetation from the background pixels, particularly for the senescent vegetation and the darkest pixels as illustrated in Supplementary Material figure S3.

#### 3.1.3. Performances of the Full SegVeg Approach

**Figure 5:**
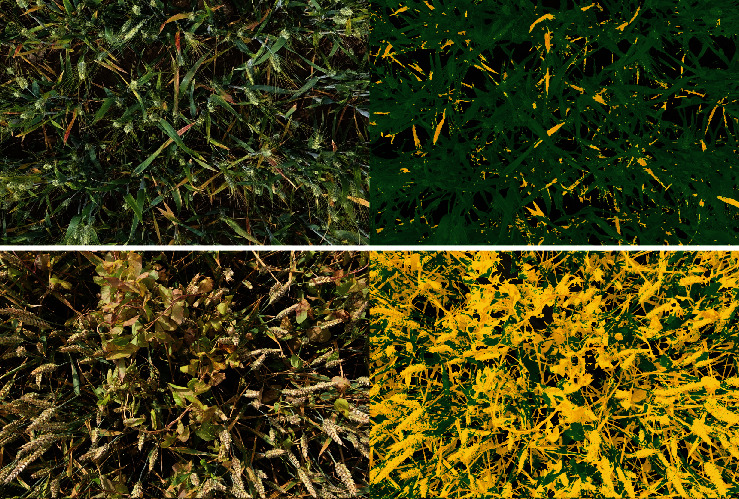
Examples of SegVeg model predictions over entire images of wheat acquired with LITERAL during early (top) and late (bottom) senescence stage. On the left, the original RGB images. On the right, the corresponding segmented images where the background, and the green and senescent vegetation are represented, respectively, in black, green, and yellow.

Results obtained over the pixels of test Dataset #2 show that the accuracy and *F*1_*all*_ score of the SegVeg model are high for the three subdatasets. The SegVeg approach classifies generally well the pixels into the three classes because of the good performances of the two stages demonstrated earlier (Table 8 and Figure 5).

**Figure 6:**
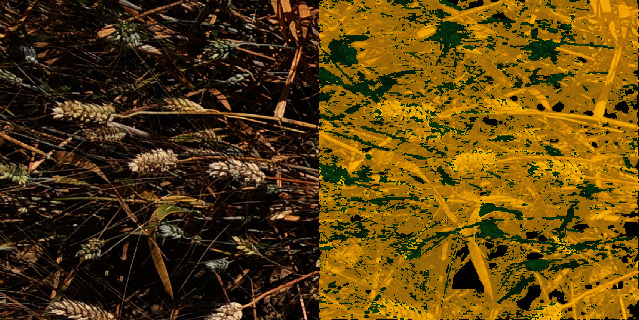
Example of misclassification with the SegVeg approach on a complex image presenting lots of thin spikes acquired with LITERAL.

However, a significant amount of misclassification is still observed between the senescent vegetation and the background for the PHENOMOBILE subdataset and between the background and the green vegetation for the LITERAL one (Figure 6). A 95% confidence interval (CI) error of 2.7 and 2.1%, respectively, for senescent vegetation and background was quantified. This CI is two times higher than that of the green vegetation class. The degraded performances observed on LITERAL images could be explained by the complexity of the images due to the presence of awns that are smaller than the pixel size, inducing confusion between classes (Figure 6).

The classification performances of SegVeg seem to slightly degrade when the green fraction decreases and when the senescent fraction increases (Supplementary Material figure S4). These situations are underrepresented in the U-net 2C training database, which may contribute to the degraded performances observed.

### 3.2. Comparison of the SegVeg Approach with the U-net 3C

Results show that U-net 3C (Table 9) performs similarly to SegVeg (Table 8) on Dataset #2.

The *Similitude* between the two models has been further studied by looking at differences in each pixel predictions between SegVeg and U-net 3C models. SegVeg pixel predictions were used as groundtruth, i.e., reference values, in confusion matrix of Table 9*Similitude* case.

The average accuracy and *F*1_*all*_ values for the *Similitude* are quite high, 90 and 85, respectively, with high values in the diagonal terms of the confusion matrix. However, on average, SegVeg approach exhibits slightly higher performances compared to U-net 3C. Tables 8 and 9 reveal that the best performances for SegVeg come mostly from a better identification of the background pixels, particularly for the LITERAL dataset.

Both models achieve the best performances on the P2S2 subdataset, whereas the worst performances are observed on the LITERAL subdataset. The poor performances are particularly due to larger confusion over the background class predicted by U-net 2C (Tables 8 and 9).

## 4. Discussion

### 4.1. Use of Different Color Spaces to Better Separate the Green and Senescent Vegetation

Differences in eye sensitivity among operators impact the perception of colors [53] and may therefore induce disagreement among them. Further, first stages of senescence may also create differences between the labelling of operators, since the yellow and reddish colors observed are in continuity with the green ones in the color space. To take into account this effect, the labelling was done using several operators to get more consensual labelling.

The colors identified as senescent vegetation during the SVM classification of the vegetation pixels show that simple thresholds in the RGB space are not sufficient to get a satisfactory separation. Reciprocally, the same applies to the green vegetation. The combined use of certain components of other color representations seem to be useful to segment the green vegetation as proposed by other authors such as R, S, a, b, Cb, and Cr in [21], sRGB space used for CIELab transformation, in [54], or H and S in [55]. Likewise, additional features may also be used to better separate the senescent vegetation such as the CMYK color space or the quadrature from YIQ that were selected as input features to the SVM (Supplementary Material figure S5).

To better highlight, qualitatively, the model performances using these features and the corresponding theoretical boundaries, a 3D RGB cube of 35^3^ voxels was created. It contains a huge panel of color shades, which helps to discern visually where the SegVeg approach locates the senescent vegetation within the color spaces (Supplementary Material figure S6).

### 4.2. Impact of Illumination Conditions on the Segmentation Performances

**Figure 7:**
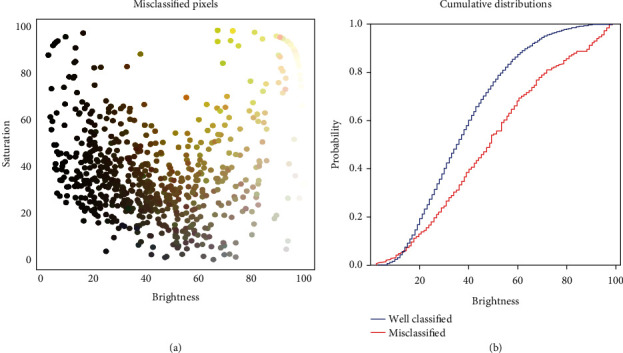
(a) Distribution of the brightness (V from HSV) and saturation (S from HSV) for the misclassified pixels by the SegVeg model. Each point corresponds to a misclassified pixel from the grids of the test dataset. They are represented by their actual RGB color. (b) Cumulated distribution of the brightness of the misclassified (red) and well-classified (blue) pixel.

The pixels misclassified by the SegVeg approach correspond mostly to brownish colors representative of the senescent vegetation or background (Figure 7(a)). The few green pixels observed with high brightness and saturation may correspond either to errors in the labelling or to mixed pixels very close to the limit between the green and senescent vegetation (Supplementary Material figure S6). Illumination conditions may also strongly impact the quality of the classification. Misclassified pixels are preferentially observed for the small brightness values (Figure 7(b)) where the dynamics of the color values may be too limited to get an accurate classification based both on the color spaces or on the spatial features, inducing confusion among the three classes. This applies both to the labelling process and to the model predictions. Misclassified pixels are also observed preferentially in the highest brightness values (Figure 7(b)). In such conditions, some authors [56] propose to assign the saturated pixels to the most frequently saturated class. In our case, this would degrade the segmentation performances since the saturated pixels may belong to any of the three classes. However, a larger representation of green vegetation particularly with glossy leaves under either clear sky conditions or using flashes is often saturated.

**Figure 8:**
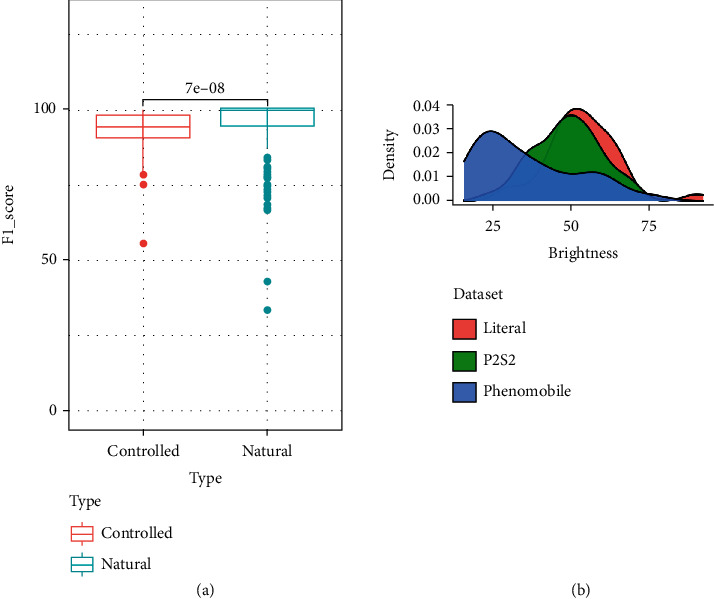
(a) Distribution of the performance (*F*1_*all*_) for both controlled (PHENOMOBILE) and natural (P2S2 and LITERAL datasets) illumination conditions (with *p* value expressed above boxplots). (b) Distribution over brightness (V from HSV) for the three datasets.

The confusions observed for the PHENOMOBILE subdataset and leading to slightly degraded segmentation performances (Figure 8(a)) are partly due to the use of flashes instead of the natural illumination as in LITERAL and P2S2 subdatasets. The noncollimated nature of the light emitted by the flashes induces a decrease in the intensity of the radiation that varies as the inverse of the square of the distance to the source. When the source is too close to the top of canopy, pixels tend to be saturated with limited classification potential. To limit this saturation effect, images taken from the PHENOMOBILE were slightly underexposed. Further, the pixels located at the bottom of the scene receive very little illumination and are therefore very dark. The distribution of the brightness for the PHENOMOBILE dataset (Figure 8(b)) shows more darker pixels than the other subdatasets acquired under natural illumination conditions. This is in agreement to the higher confusion between the vegetation and the background presented earlier (Tables 7 and 8).

### 4.3. Weak Supervision Is Promising

**Figure 9:**
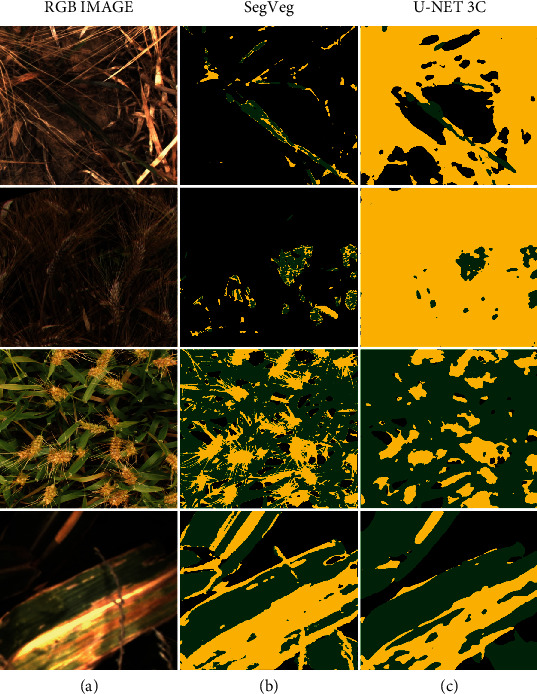
Results of the segmentation using SegVeg (b) or U-net 3C (c). Background, green vegetation, and senescent vegetation are represented, respectively, in black, green, and yellow. (a) The original RGB image.

Because of the unavailability of images fully labelled into the three classes, U-net 3C was trained over masks predicted by the SegVeg model. This weak supervision approach could lead to biased predictions, since SegVeg predicted masks are not perfect as demonstrated previously in Table 8, and obviously, training will converge to similar SegVeg results. Moreover, U-net 3C was trained over whole images compared to 6132 pixels for SVM classification model. However, the performances of U-net 3C (Table 9) are quite close to those of SegVeg (Table 8) for the PHENOMOBILE and P2S2 subdatasets, while SegVeg performs slightly better over the LITERAL subdataset. Comparison between SegVeg and U-net 3C (Table 9, “Similitude” case) confirms the consistency between the two models, as expected. Weak supervision appears to be a promising way to pretrain deep learning algorithms by reducing the labelling process by the operators. The larger number of images therefore available to train the model is expected to partly compensate for the lower quality of the “automatic” labelling. However, the main differences lie in the patterns of the green and senescent vegetation masks (Figure 9) where SegVeg appears crisper than U-net 3C which shows fuzzier masks. Indeed, the kernel filters used in U-net 3C to separate the green from the senescent vegetation tend to omit the small elements in the images and render more diffused patches. Conversely, the pixel-based separation between the green and senescent vegetation allows to better describe the small details (Figure 9).

### 4.4. Predicting the Fractions of Green and Senescent Vegetation

The evaluation of the performances over pixels that have been labelled by the operators has been presented. However, the grid-pixels correspond to a subsample of the image which questions their representativeness in regard to the entire image. We therefore evaluated the agreement between the segmentation predicted by SegVeg and by U-net 3C over both the grid-pixels and the entire images, following the same exact principle as Table 9, SegVeg pixels as reference. Results show (Table 10, “Similitude” case) that *R*^2^, RMSE, slope, and offset for the grids and the images are in good agreement of each of the three fractions considered. This indicates the fraction of background, green, and senescent vegetation computed over the pixel subsampling represents quite well the whole images.

**Figure 10:**
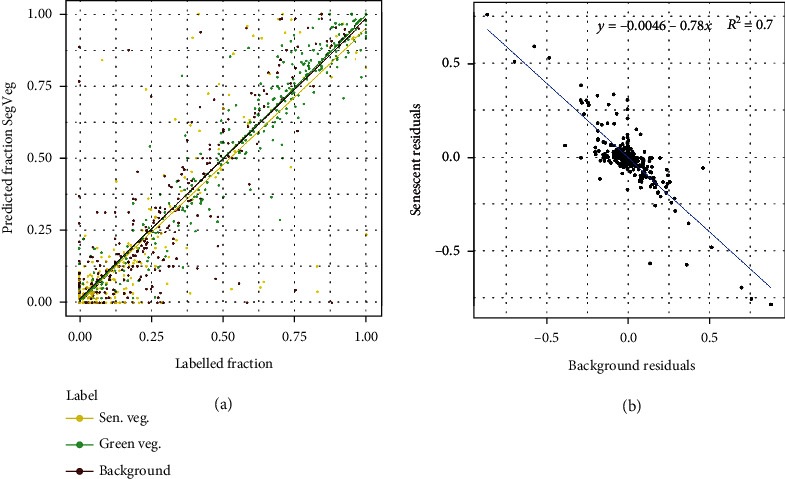
(a) Comparison between the fractions predicted by the SegVeg approach and the labelled ones over test Dataset #1: green vegetation (green), senescent vegetation (yellow), and background (brown). The best fit for each fraction is represented by a solid line of the same color. (b) Relationship between the residual of the background and senescent fractions.

SegVeg and U-net 3C show similar performances. The best agreement is observed for the green vegetation fraction (Table 10), with a slight advantage for SegVeg, confirming the slightly better performances in the segmentation of this class (Tables 8 and 9). The estimates are not biased, according to slopes (Table 8 and Figure 10(a)). Conversely, the estimation of the background and senescent vegetation fractions show degraded performances for U-net 3C, which are related to the degraded performances observed previously in the segmentation of these two classes. The confusion between the background and the senescent vegetation pixels by U-net 3C may be quite large as highlighted by the number of outliers, with a quasiexact compensation between these two fractions since the green vegetation fraction is well predicted (Figure 10(b)). Small biases are observed in these fractions predicted by SegVeg and U-net 3C models, except for the senescent fraction of U-net 3C for which the bias (Table 10) mostly comes from the distribution of the outliers (Figure 10).

The SegVeg approach and U-net 3C segmentation appear efficient to compute the fractions of the different elements of the image. However, the SegVeg model offers a slight advantage with better performances for green fraction and smaller biases in senescent vegetation fraction.

### 4.5. Limitation of the Study

This study is based on segmentation models using shallow and deep learning techniques. It is therefore constrained by the availability of training and testing datasets. The first-stage SegVeg U-net 2C model was trained over a relatively large and diverse database (Table 1) containing 2015 images of 512 × 512 pixels. The SegVeg SVM is trained over 6132 pixels extracted from grids applied to the original images, thus showing a wide diversity in species, phenological stages, canopy state, and acquisition conditions. However, the pixels labelled as uncertain (green/Sen. Veg. unsure, unknown, and other) were not used, forcing the SVM model to extrapolate for these situations. Finally, the training was completed over two subdatasets where the P2S2 is overrepresented as compared to PHENOMOBILE. This is why the results were presented per subdataset. This also partly explains the differences in performances observed over the three test datasets, with a general trend: P2S2 > PHENOMOBILE > LITERAL.

The evaluation of the models was performed at the pixel level. A large number of pixels was considered here (more than 20,000 pixels, including background class, Table 5), along with those extracted from the LITERAL subdataset that were not used in training. The “unsure” pixels were not used to compute the performances, which may also induce small biases in the results since the “unsure” pixels may not be evenly distributed between the three classes of interest. However, we did not have other alternatives, since “unsure” pixels correspond mostly to extremely dark, bright (S3), or mixed pixels. Indeed, great attention should be paid to the image spatial resolution and exposure during image acquisition. Studies based on 3D scenes rendered realistically should be conducted to better understand the unsure classes and their possible distribution among the three classes of interest.

## Figures and Tables

**Table 1:** Characteristics of the subdatasets composing the final dataset.

Subdatasets	Country	Year	Crops	Stage	Reference
UTokyo	Japan	2019	Rice, wheat	Vegetative	[40, 41]
		2012			
P2S2	France	2018	Wheat, rapeseed, sugar beet, and potato	All	[42]
	Belgium		Maize, grassland, sunflower, rice, and soya		
Wuhan	China	2012	Cotton, maize, and rice	Vegetative	[43]
		2015			
CVPPP 1 and 2	Italy	2012	Arabidopsis, tobacco	All	[44]
		2013			
GEVES	France	2020	Maize	Vegetative	—
Phenofix	France	2020	Maize	All	—
Phenomobile	France	2020	Wheat	Early	—
Bonirob	Germany	2016	Sugar beet	Early	[45]

**Table 2:** Characteristics of the subdatasets used to compose the training dataset. UGV means unmanned ground vehicle.

Subdatasets	Platform	Camera	Image size (px)	Distance to ground (m)	GSD (mm)	No. of images
UTokyo	Gantry	Canon EOS Kiss X5	5184 × 3456	1.5-1.8	0.2-0.6	534
		Garden Watch Camera	1280 × 1024			
P2S2	Handheld	SONY ILCE-5000	5456 × 3632	2	0.5	170
		SONY ILCE-6000	6000 × 5000			
		Canon EOS 400D	3888 × 2592			
		Canon EOS 60D	5184 × 3456			
		Canon EOS 750D	6000 × 4000			
Wuhan	Gantry	Olympus E-450	3648 × 2736	0.3-5	0.4-0.5	343
CVPPP 1 & 2	Gantry	Canon PowerShotSD1000	3108 × 2324	1	0.1-0.3	752
GEVES	Handheld	SAMSUNG SM-A705FN	3264 × 1836	2	0.2	50
Phenofix	Gantry	SONY RX0 II	4800 × 3200	2	0.6	30
Phenomobile	UGV	SONY RX0 II	4800 × 3200	1.7	0.8-1.4	76
Bonirob	UGV	JAI AD-130GE	1296 × 966	0.85	0.3	60
Total						2015

**Table 3:** Second-stage dataset description.

Datasets	LITERAL	PHENOMOBILE	P2S2
Latitude, longitude	43.7° N, 5.8° E 49.7° N, 3.0° E 43.5° N, 1.5° E	43.7° N, 6.7° E 47.4° N, 2.3° E 43.7° N, 5.8° E 43.4° N, 0.4° W	43.6° N, 4.5° E 43.4° N, 1.2° E 48.3° N, 2.4° E 50.6° N, 4.7° E
Year	2017-2020	2018-2020	2018
Crops	Wheat	Wheat, sunflower, sugar beet, maize, potato, and flax	Wheat, sunflower, sugar beet, maize, potato, rapeseed, grassland, rice, and soya
Vector	Handheld	Phenomobile	Handheld
Focal length (mm)	8	16–25	50
Camera	Sony RX0 II	Baumer VCXG-124C	ILCE-6000 SONY Canon EOS 750D
Image size (pixels)	4800 × 3200	4096 × 3000	6000 × 4000 3888 × 2592
Pixel size (*μ*m)	2.74	3.45	3.72
Distance to ground (m)	1.5–2.5	2–4.5	1.5–2
GSD (mm)	0.65	1.3	0.5

**Table 4:** Distribution of labelled pixel for the three datasets.

Datasets	No. of labelled images	No. of labelled pixels	% classes					
			Green veg.	Sen. Veg.	Background	Green/Sen. Veg. unsure	Unknown	Other
LITERAL	68	4260	46.5	15.8	15.0	13.1	9.5	0.1
PHENOMOBILE	173	8266	40.3	31.1	27.6	0.1	0.8	0.1
P2S2	200	18559	43.6	16	15.2	11.1	13	1.1
Total	441	31085	43.4	20.5	19.75	8.1	7.8	0.45

**Table 5:** Distribution of the labelled pixels into the training and testing datasets. Only the pixels labelled as *Green Veg.* and *Sen. Veg.* were used for the SVM SegVeg training.

Datasets	No. of labelled pixels	% classes		No. of pixels train	No. of pixels test	% train	% test
		Green Veg.	Sen. Veg.				
LITERAL	2655	75	25	0	2655	0	100
PHENOMOBILE	5883	60	40	1803	4080	30	70
P2S2	11200	75	25	4329	6871	39	61
Total	19738	70	30	6132	13606	32	68

**Table 6:** Metrics used to evaluate the performances of the models.

Metrics	Name	Definition
True positive	Tp_*class*_	Number of pixels well predicted in the given class
True negative	Tn_*class*_	Number of pixels well predicted as not in the given class
False positive	Fp_*class*_	Number of pixels wrongly predicted in the given class (confusion)
False negative	Fn_*class*_	Number of pixels wrongly predicted as not in the given class (missing pixels)
Precision	Prec_*class*_	*Tp*/*Tp* + *Fp*
Recall	Rec_*class*_	*Tp*/(*Tp* + *Fn*)
Accuracy	Acc_*class*_	((*Tp* + *Tn*)/(*Tp* + *Tn* + *Fp* + *Fn*)) × 100
*F*1-score	F1_*class*_	((2 × *Tp*)/(2 × *Tp* + *Fp* + *Fn*)) × 100
Overall *F*1-score	F1_*All*_	(1/*N*)∑_*i*=0_^*N*^*F*1 − *score*_*i*_ × 100
% confidence interval error	CI	$1.96\times \sqrt{\left(F1_{score}\times \left(1-F1_{score}\right)\right)/n}$
RMSE	RMSE	$\sqrt{\left(1/n\right)\sum_{i=1}^{n}{\left({y_{i}}^{theorical}-{y_{i}}^{predicted}\right)}^{2}}$
*R* ^2^	*R* ^2^	1 − ((∑(*y*_*i*_^*predicted*^ − *y*_*i*_^*theorical*^)^2^)/(∑(*y*_*i*_^*theorical*^ − *y*_*i*_^*mean*^)^2^))
Canopy fraction	CF_class	∑_*i*=1_^*I*^∑_*j*=1_^*J*^(*image*(*i*, *j*) = *class*)/∑_*i*=1_^*I*^∑_*j*=1_^*J*^(*image*(*i*, *j*)) (where *i* and *j* are, respectively, the width and height of the image in pixels)

**Table 7:** Performances of the U-net 2C model to classify vegetation (*Green* *Veg*.+*Sen*.*Veg*.) and background (Back.) pixels over test Dataset #2. The elements of the confusion matrix, *F*1*class* and *F*1_*all*_, are presented.

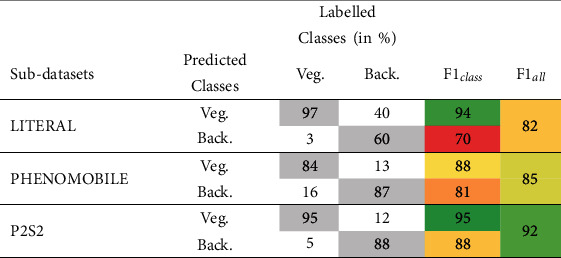

**Table 8:** Confusion matrix (in % of the labelled pixels), accuracy, and *F*1_*all*_ values computed for the SVM classification only and using the full SegVeg approach for the three subdatasets (e.g., pixels from Dataset #2). The diagonal terms of the confusion matrix are indicated in gray color. The colors of the two last columns correspond to the accuracy and *F*1_*all*_ values (dark green, highest; dark red, lowest).

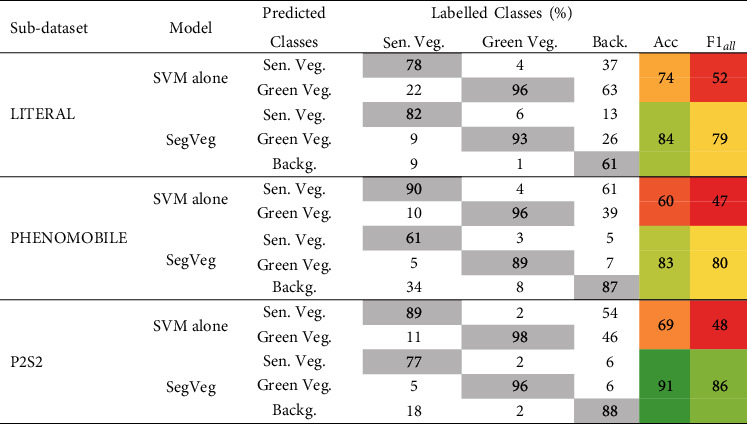

**Table 9:** Performances of U-net 3C model and Similitude to SegVeg model evaluation (in %). Similitude confusion matrix was built with SegVeg outputs as groundtruth pixel values on Dataset #2. The diagonal terms of the confusion matrix are indicated in gray cells. The colors of accuracy and *F*1_*all*_ are related to their performances (dark red the lowest; dark green the highest).

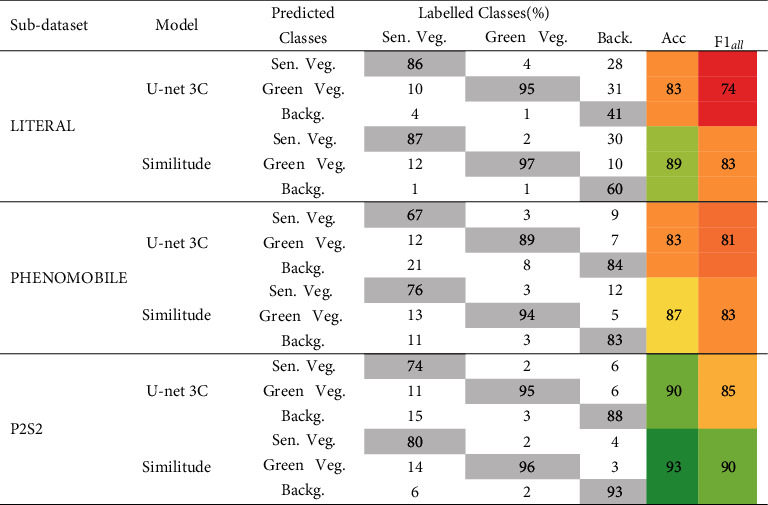

**Table 10:** Performances of SegVeg and U-net 3C to estimate the background, green, and senescent vegetation fractions over grids. “Similitude” for comparison of model performances was computed using either the labelled grids or whole images. *R*^2^ is the determination coefficient. The colors of *R*^2^ and RMSE are related to their column values (dark green, the best; dark red, the worst).

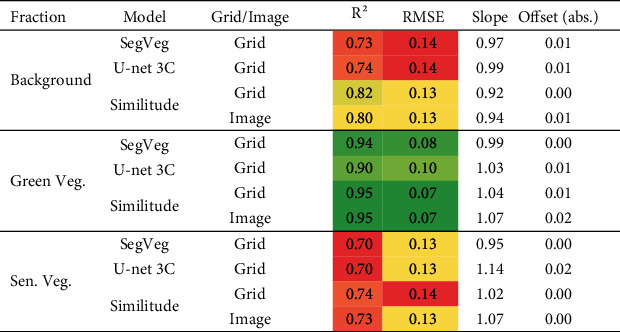

## Data Availability

SegVeg pixels dataset, images, and their corresponding segmentation masks are be publicly available. All the SegVeg scripts for computation and analysis are also public: https://github.com/mserouar/SegVeg. For simplicity, dataset download links (including Zenodo) will be specified in the above repository.

## Supplementary Materials

Supplementary 1 Video record of grid-pixel annotation process. The annotator classifies every pixel at the intersection of the grid to the one of the six classes.

Supplementary 2 Example of classification errors with the vegetation and background first-stage U-net 2C model. Left: original images. Middle: vegetation masked images. Right: background masked images. Top: almost all the senescent vegetation is classified as soil. Bottom: background algae zones are classified as vegetation.

Supplementary 3 Distribution of the colors among the six classes as observed over the labelled pixels of the test and training datasets. For each class, pixels are sorted according to their brightness from the HSV color space.

Supplementary 4 Performances (*F*1 all) of the SegVeg approach as a function of the green (left) and senescent fraction (right) per image.

Supplementary 5 Use of different meaningful color spaces to describe the image content: RGB original image (left), Y component from CMYK (middle), and Q component from YIQ (right). Y and Q images are in gray scale.

Supplementary 6 Boundaries of SegVeg colors inferred on a 35 3-voxel RGB cube thanks to the SegVeg model second-stage SVM. On the right, yellow predicted pixels. On the left, the rest that includes the green predicted pixels.
