# Supplementary figures and images for: SegVeg: Segmenting RGB Images into Green and Senescent Vegetation by Combining Deep and Shallow Methods
Source: Plant Phenomics. 2022 Oct 11;2022:9803570. doi: 10.34133/2022/9803570 (PMC9680505; doi:10.34133/2022/9803570)

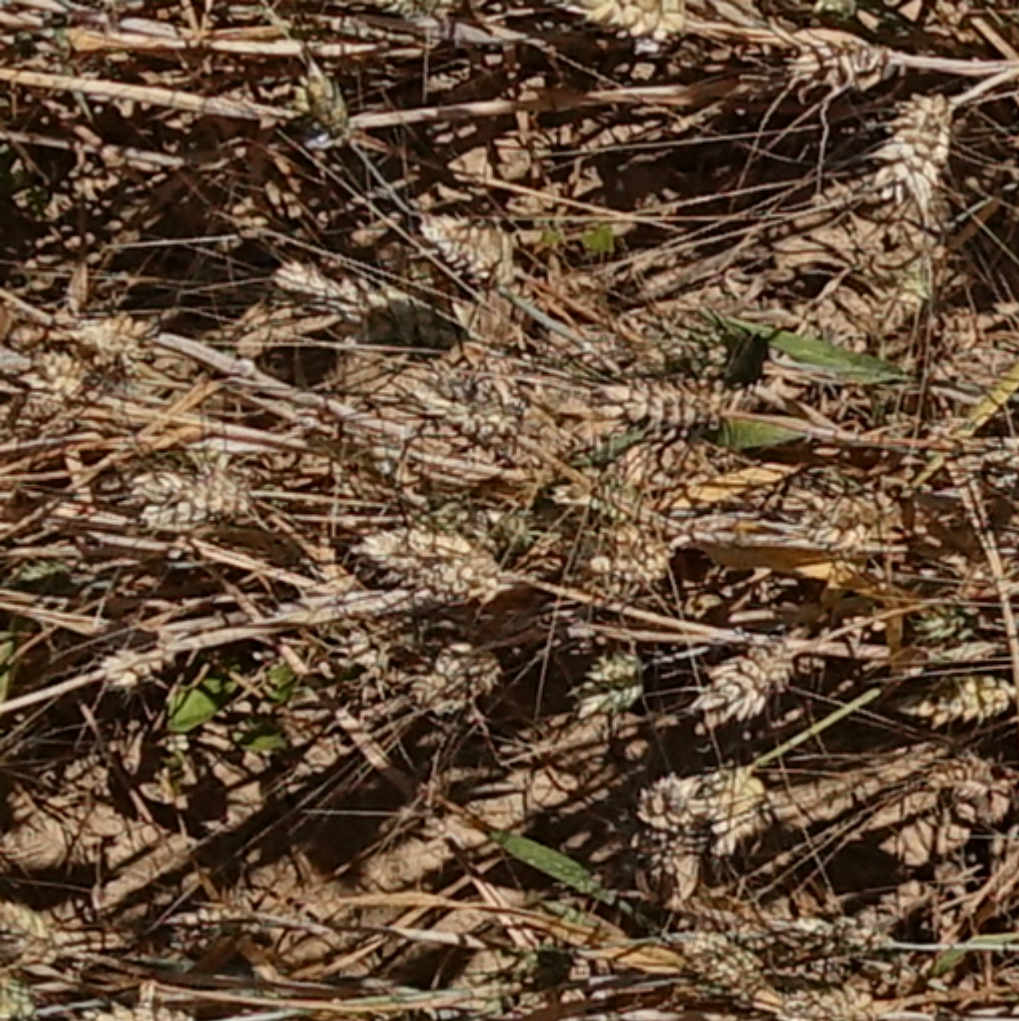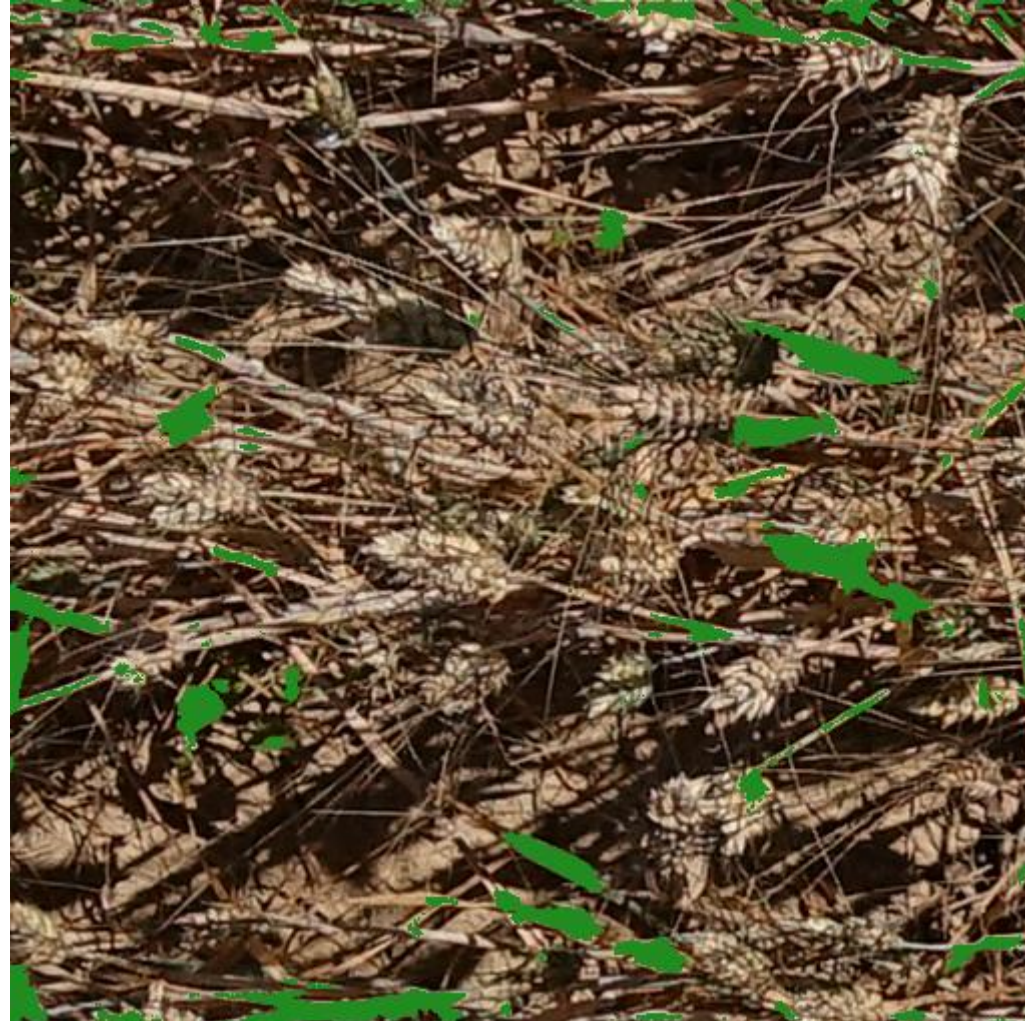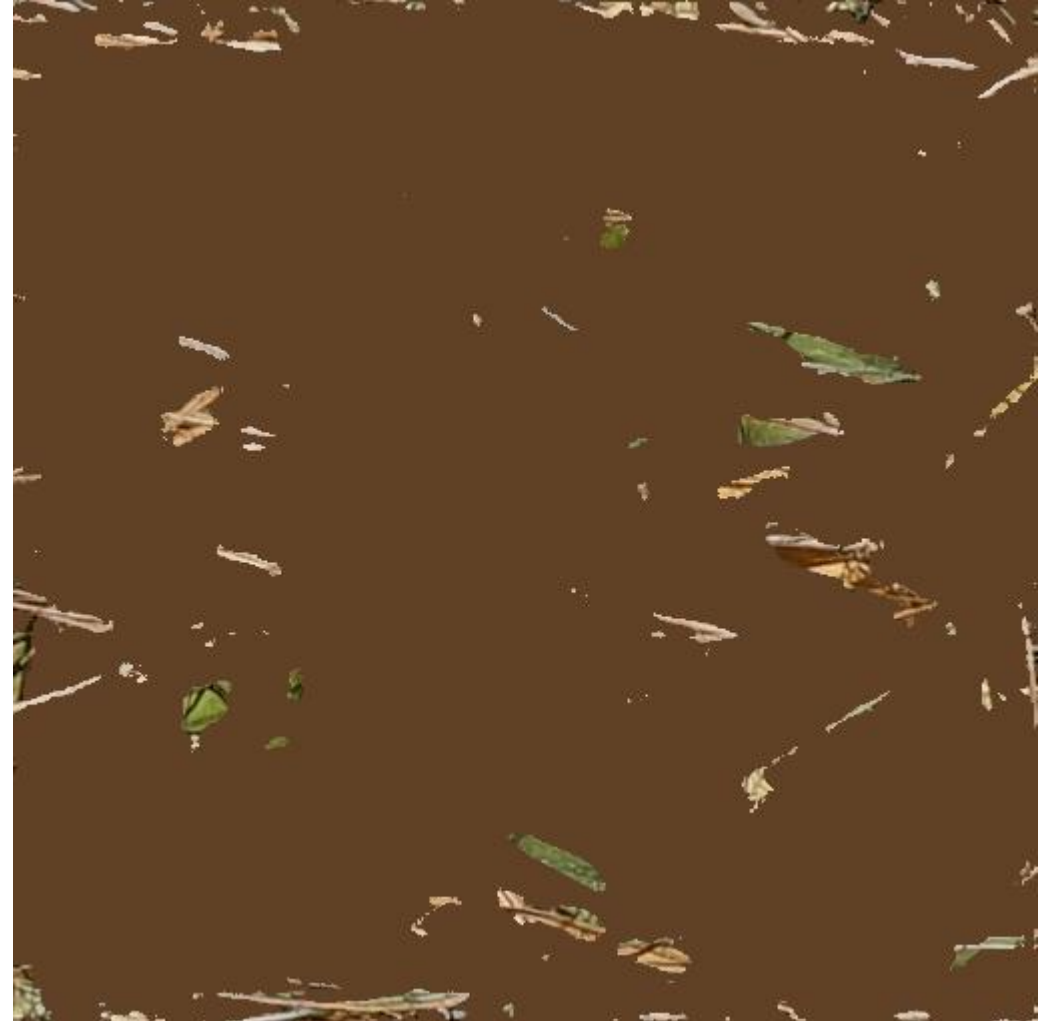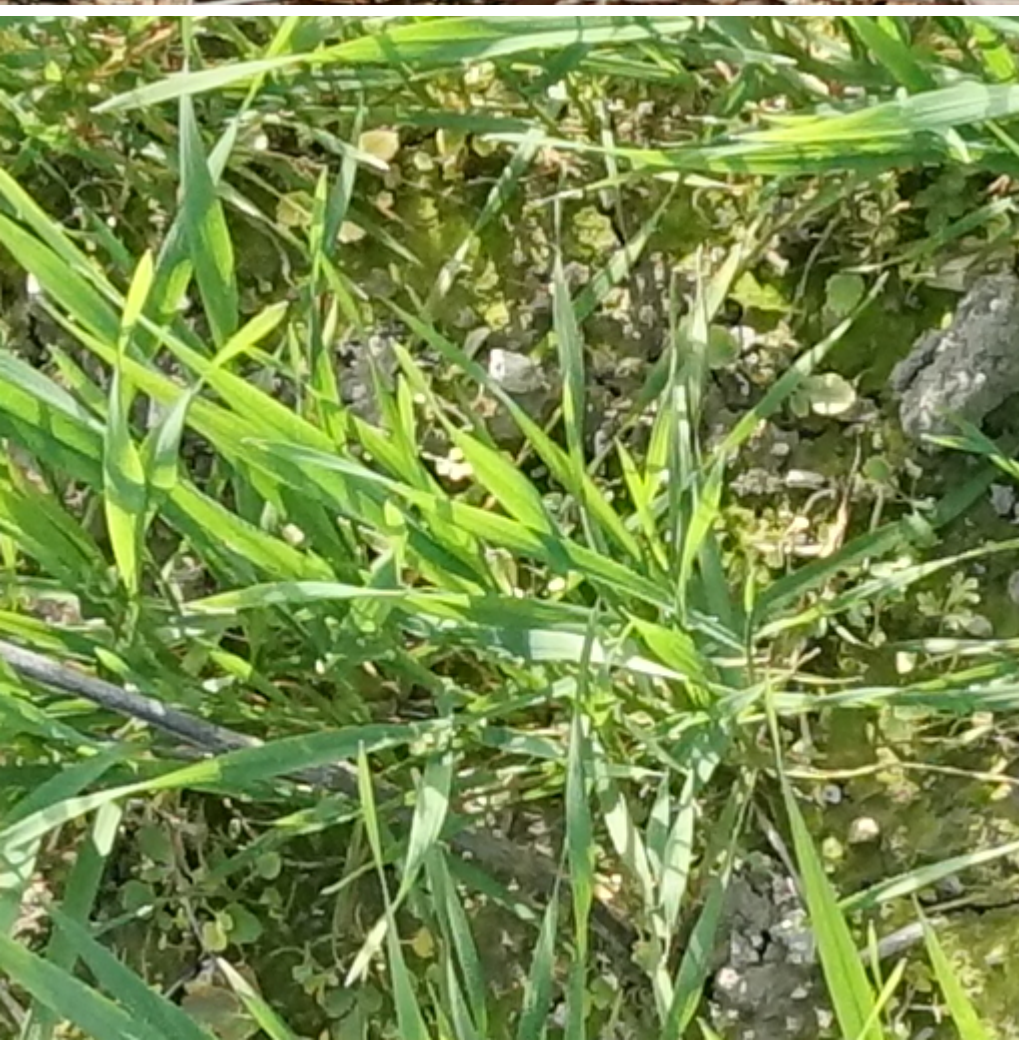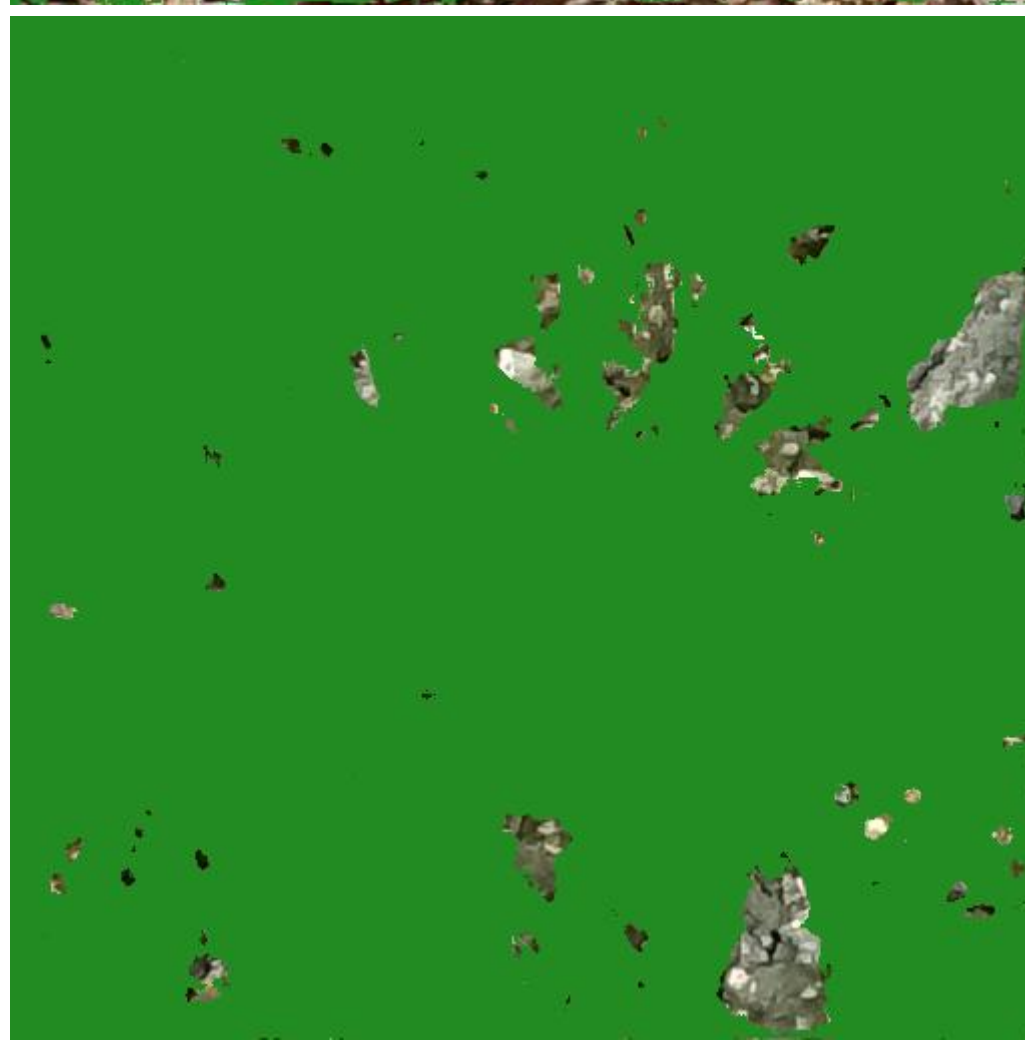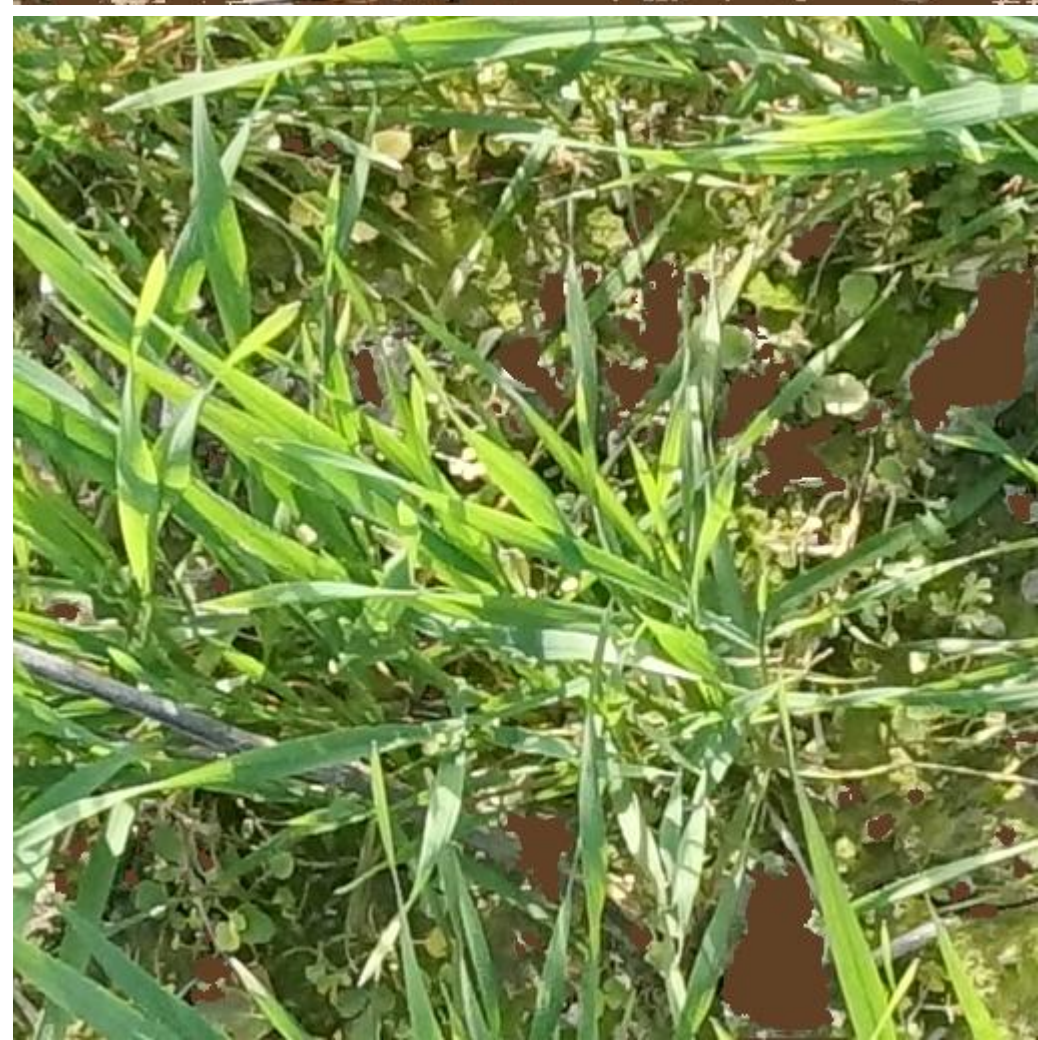

Supplement: Supplementary 2 — Example of classification errors with the vegetation and background first-stage U-net 2C model. Left: original images. Middle: vegetation masked images. Right: background masked images. Top: almost all the senescent vegetation is classified as soil. Bottom: background algae zones are classified as vegetation. [file 9803570.f2.pdf]

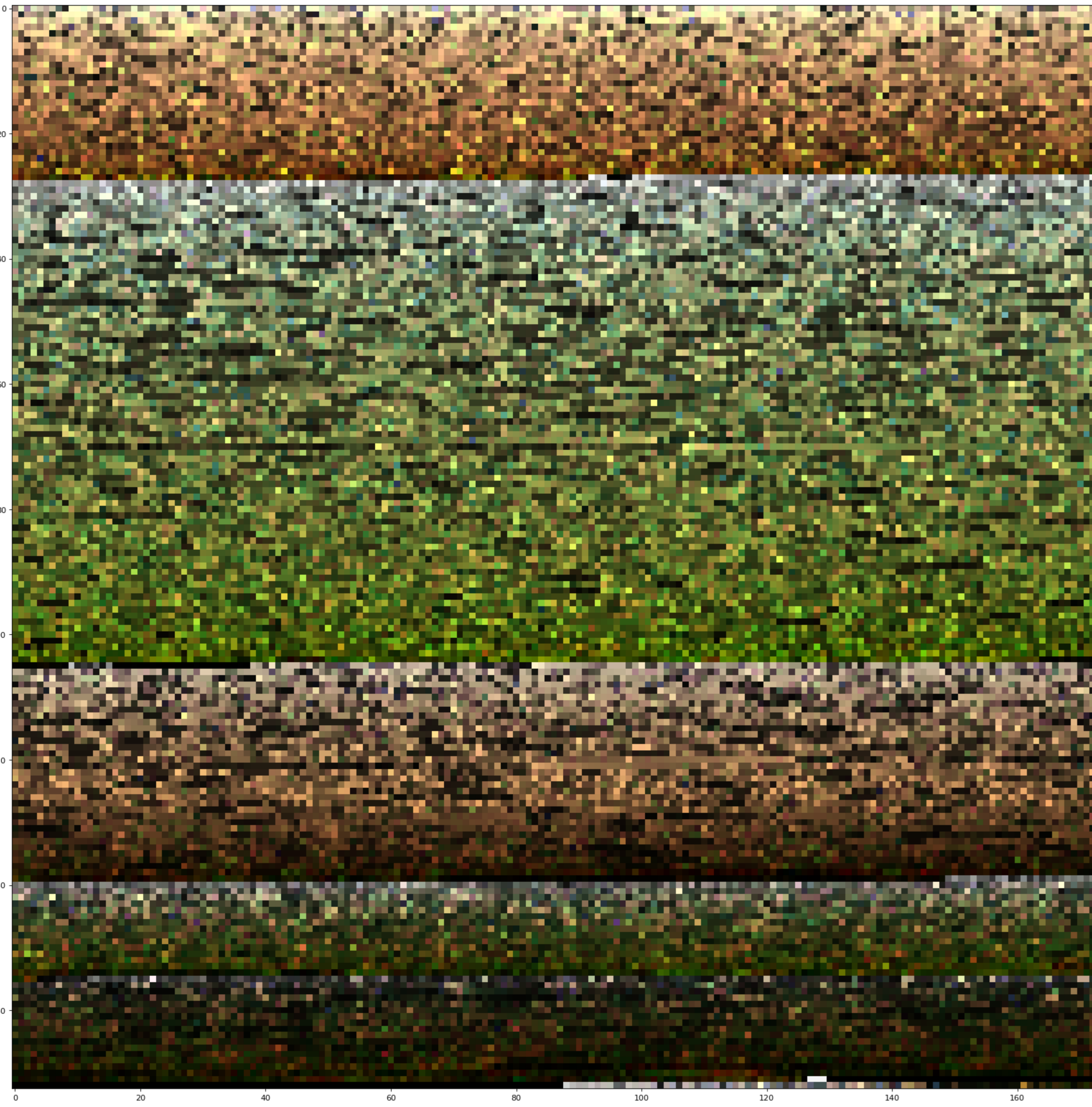

Senescent Vegetation

Green Vegetation

Background

Green / Senescent Veg.  
Unsure

Unknown

Other

Supplement: Supplementary 3 — Distribution of the colors among the six classes as observed over the labelled pixels of the test and training datasets. For each class, pixels are sorted according to their brightness from the HSV color space. [file 9803570.f3.pdf]

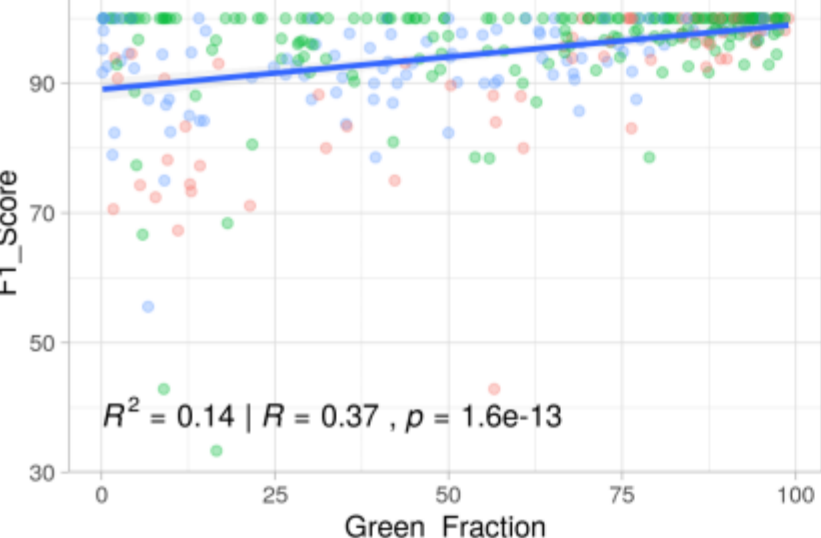

#### DATASET

- LITERAL
- P2S2
- PHENOMOBILE

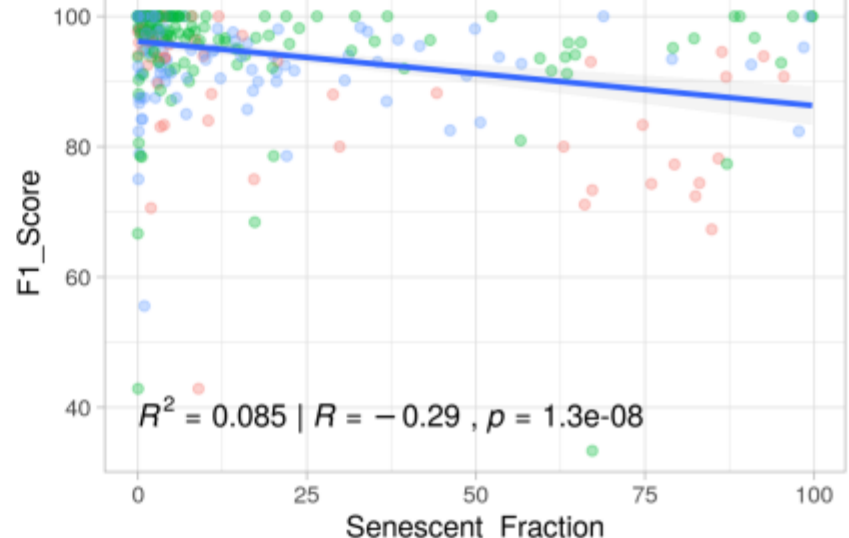

Supplement: Supplementary 4 — Performances (F1 all) of the SegVeg approach as a function of the green (left) and senescent fraction (right) per image. [file 9803570.f4.pdf]

RGB IMAGE

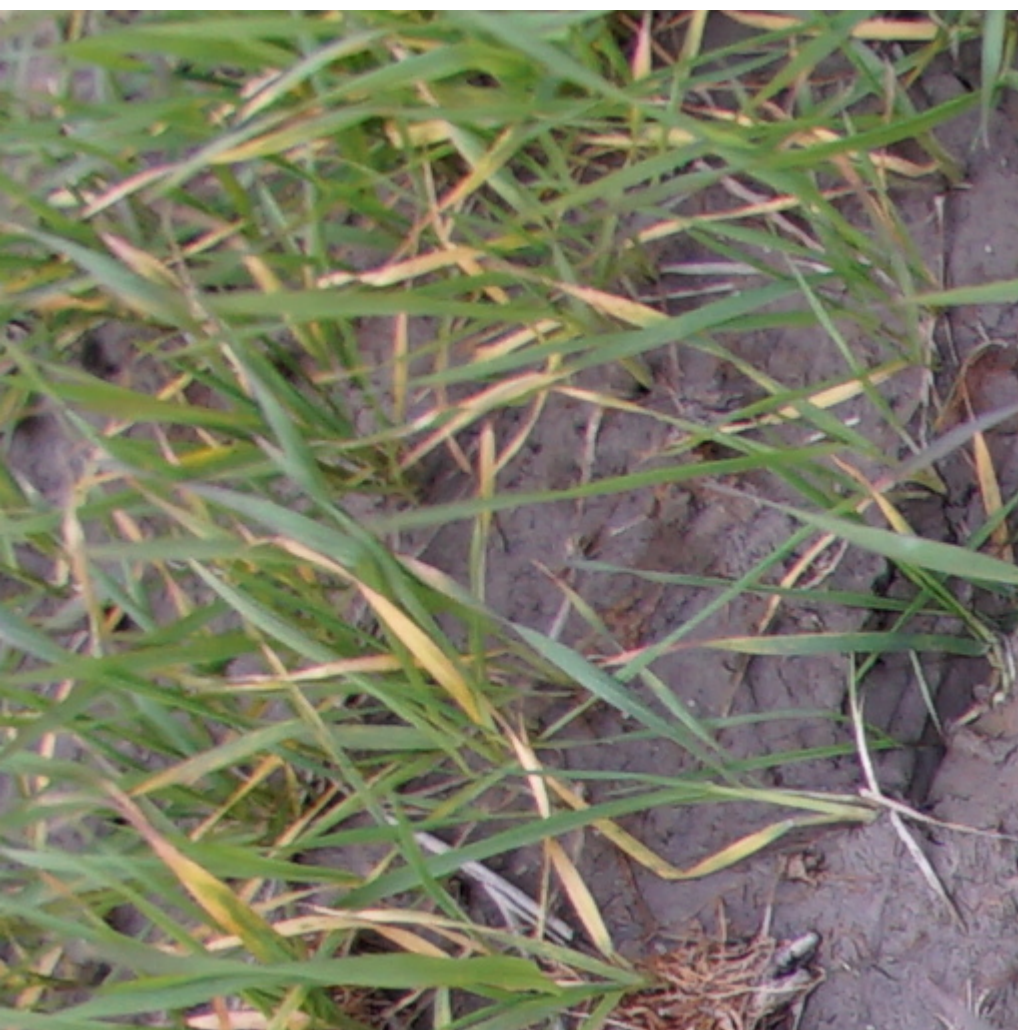

Y (from CMYK)

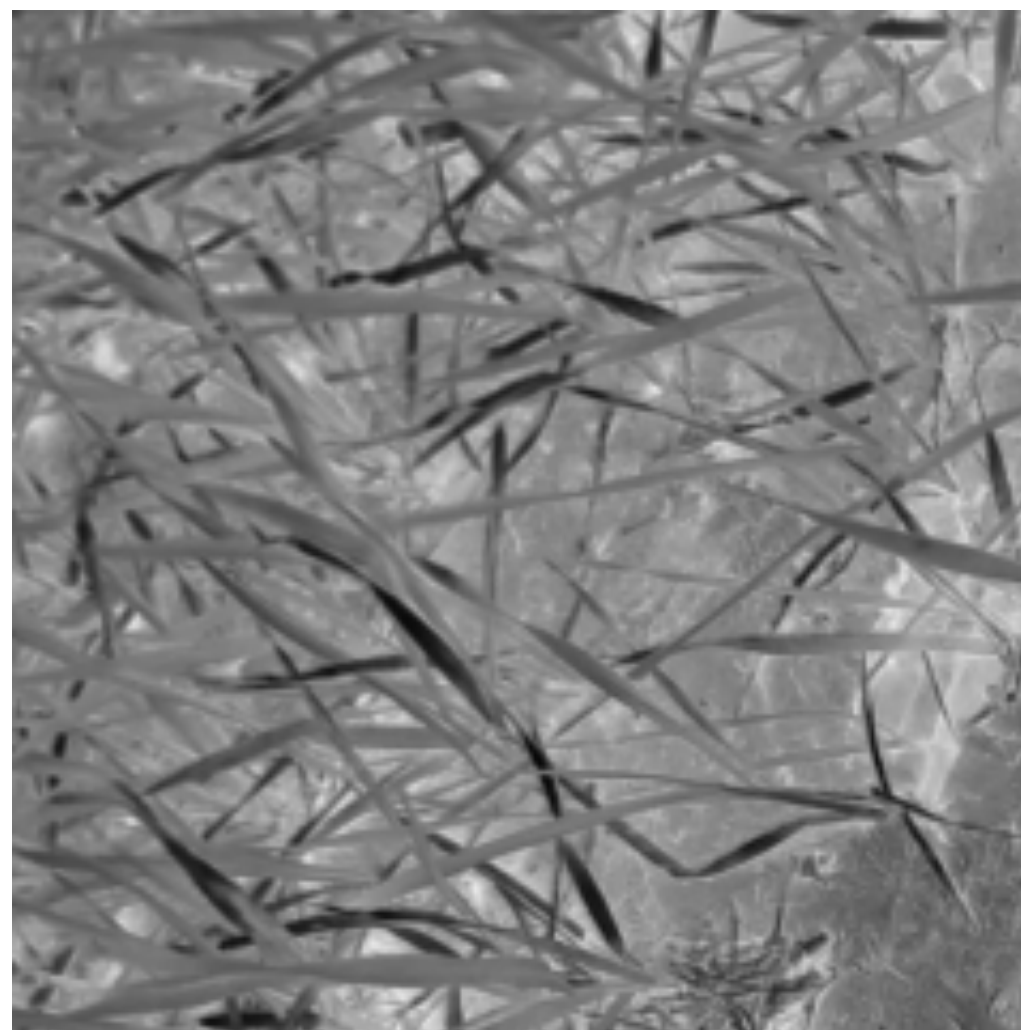

Q (from YIQ)

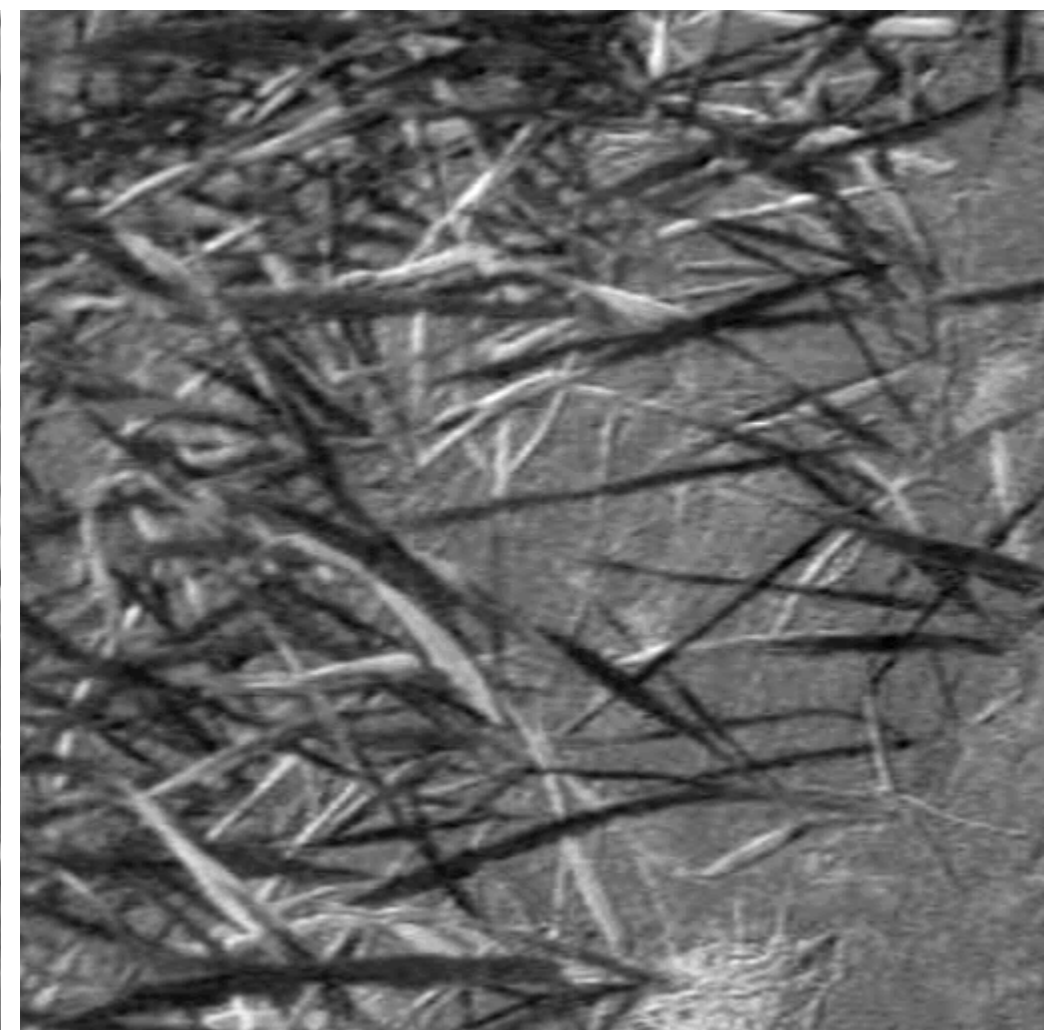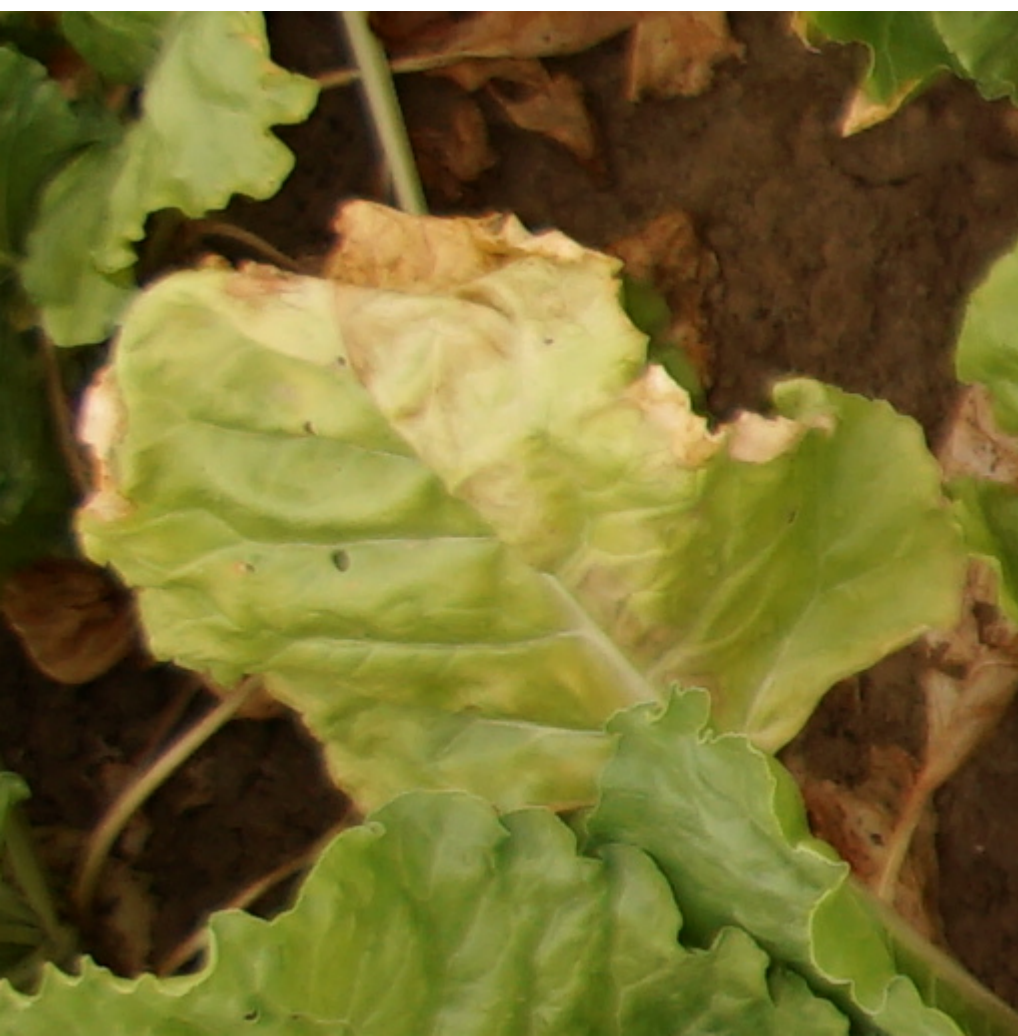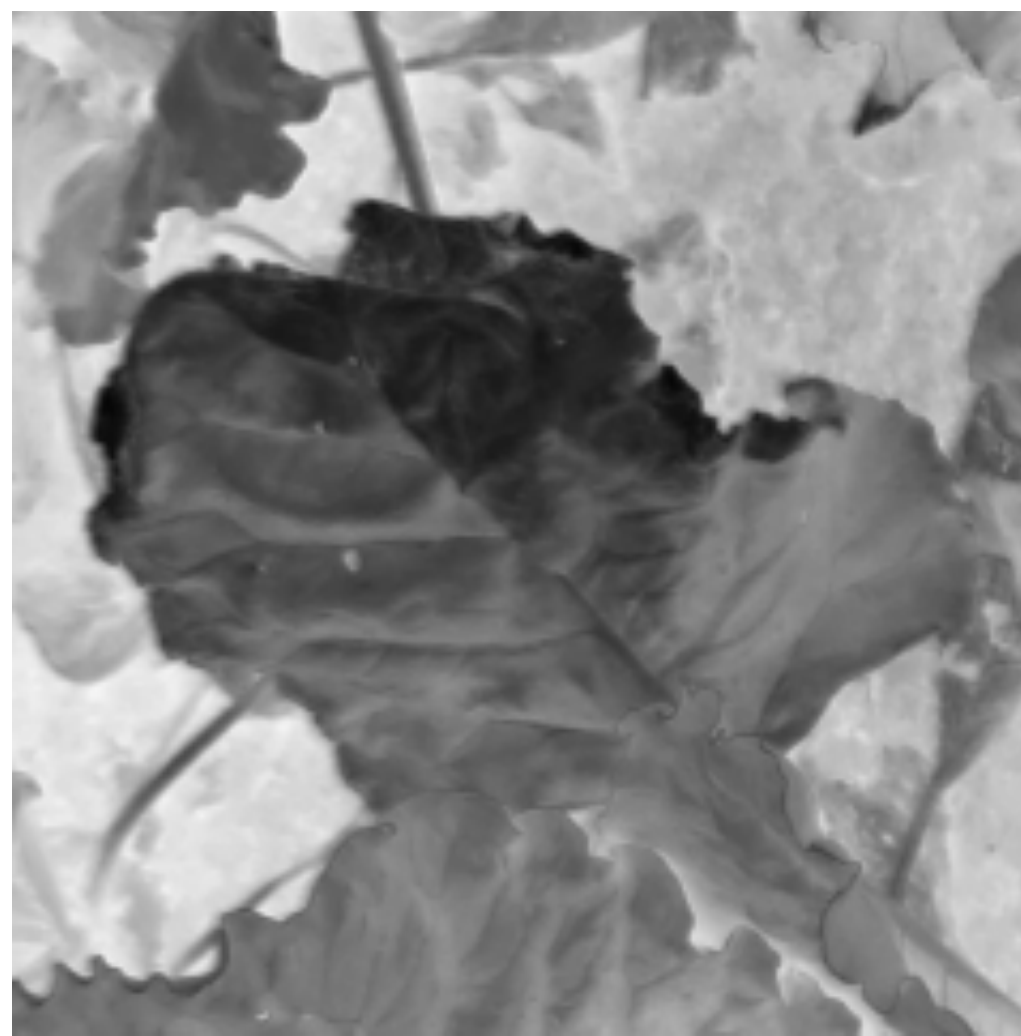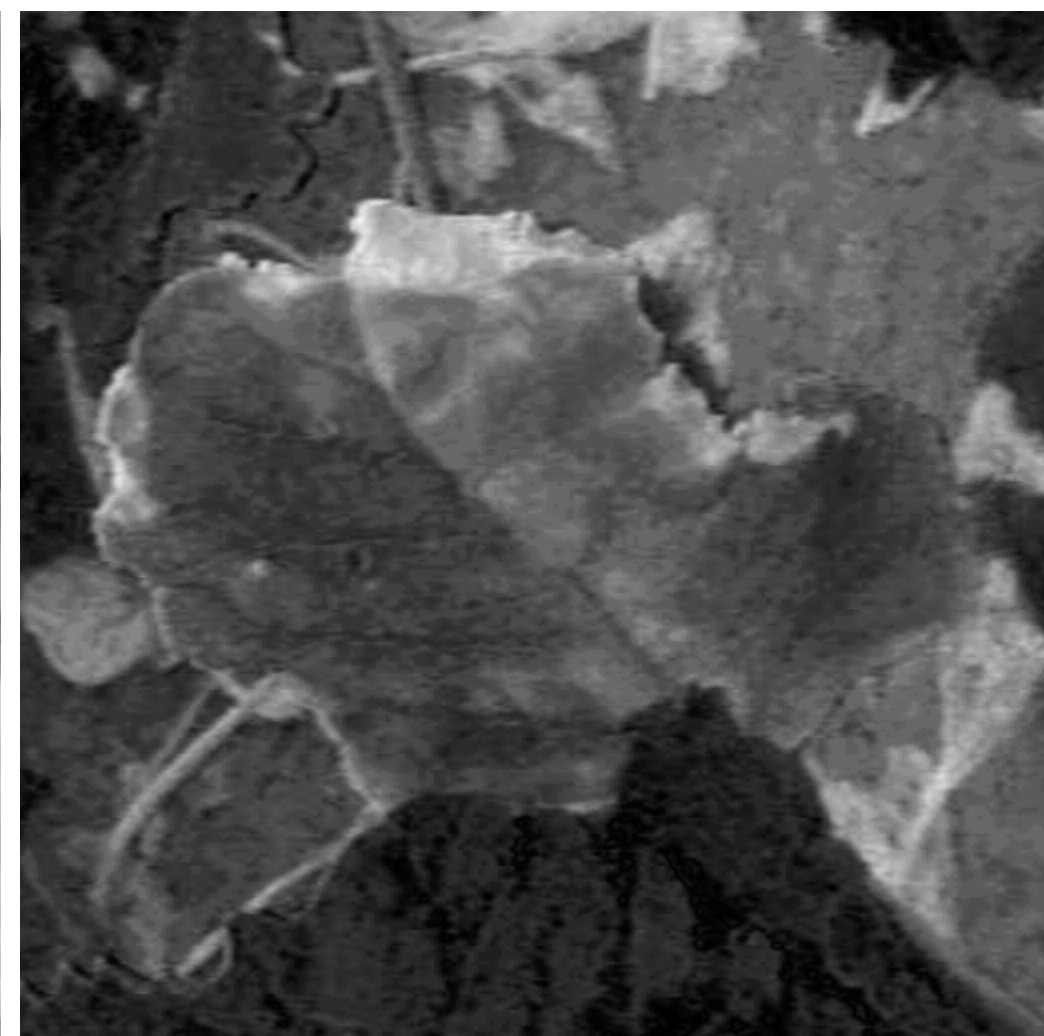

Supplement: Supplementary 5 — Use of different meaningful color spaces to describe the image content: RGB original image (left), Y component from CMYK (middle), and Q component from YIQ (right). Y and Q images are in gray scale. [file 9803570.f5.pdf]

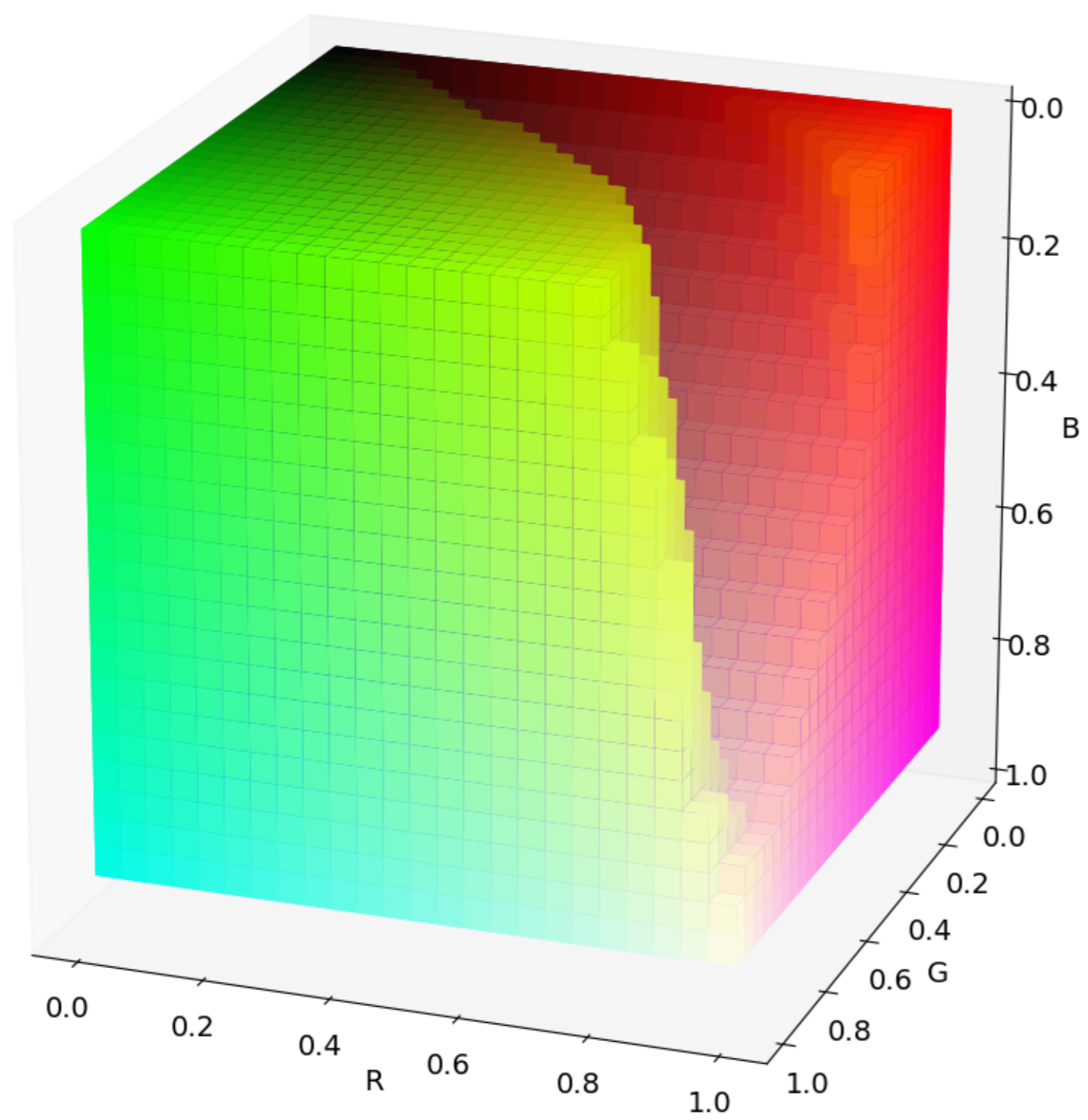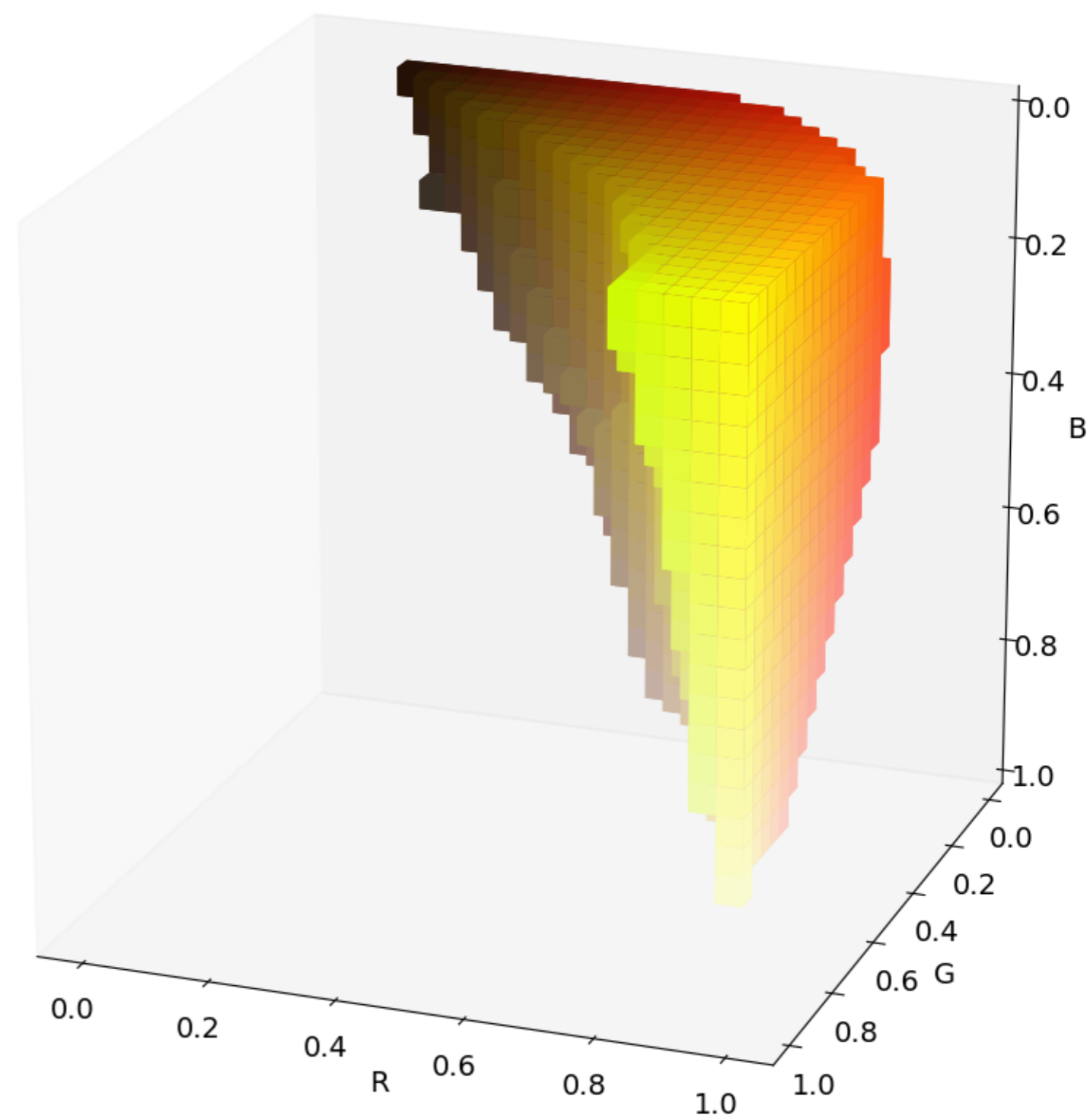

Supplement: Supplementary 6 — Boundaries of SegVeg colors inferred on a 35 3-voxel RGB cube thanks to the SegVeg model second-stage SVM. On the right, yellow predicted pixels. On the left, the rest that includes the green predicted pixels. [file 9803570.f6.pdf]
